# Genome-scale model development and genomic sequencing of the oleaginous clade *Lipomyces*

**DOI:** 10.3389/fbioe.2024.1356551

**Published:** 2024-04-04

**Authors:** Jeffrey J. Czajka, Yichao Han, Joonhoon Kim, Stephen J. Mondo, Beth A. Hofstad, AnaLaura Robles, Sajeet Haridas, Robert Riley, Kurt LaButti, Jasmyn Pangilinan, William Andreopoulos, Anna Lipzen, Juying Yan, Mei Wang, Vivian Ng, Igor V. Grigoriev, Joseph W. Spatafora, Jon K. Magnuson, Scott E. Baker, Kyle R. Pomraning

**Affiliations:** ^1^ Energy and Environment Directorate, Pacific Northwest National Laboratory, Richland, WA, United States; ^2^ US Department of Energy Agile BioFoundry, Emeryville, CA, United States; ^3^ US Department of Energy Joint BioEnergy Institute, Emeryville, CA, United States; ^4^ US Department of Energy Joint Genome Institute, Lawrence Berkeley National Laboratory, Berkeley, CA, United States; ^5^ Department of Agricultural Biology, Colorado State University, Fort Collins, CO, United States; ^6^ Environmental Genomics and Systems Biology Division, Lawrence Berkeley National Laboratory, Berkeley, CA, United States; ^7^ Department of Plant and Microbial Biology, University of California, Berkeley, Berkeley, CA, United States; ^8^ Department of Botany and Plant Pathology, Oregon State University, Corvallis, OR, United States; ^9^ Earth and Biological Sciences Directorate, Pacific Northwest National Laboratory, Richland, WA, United States

**Keywords:** oleaginous yeasts, genome-scale metabolic model, flux balance analysis, *Lipomyces*, genome sequencing

## Abstract

The *Lipomyces* clade contains oleaginous yeast species with advantageous metabolic features for biochemical and biofuel production. Limited knowledge about the metabolic networks of the species and limited tools for genetic engineering have led to a relatively small amount of research on the microbes. Here, a genome-scale metabolic model (GSM) of *Lipomyces starkeyi* NRRL Y-11557 was built using orthologous protein mappings to model yeast species. Phenotypic growth assays were used to validate the GSM (66% accuracy) and indicated that NRRL Y-11557 utilized diverse carbohydrates but had more limited catabolism of organic acids. The final GSM contained 2,193 reactions, 1,909 metabolites, and 996 genes and was thus named iLst996. The model contained 96 of the annotated carbohydrate-active enzymes. iLst996 predicted a flux distribution in line with oleaginous yeast measurements and was utilized to predict theoretical lipid yields. Twenty-five other yeasts in the *Lipomyces* clade were then genome sequenced and annotated. Sixteen of the *Lipomyces* species had orthologs for more than 97% of the iLst996 genes, demonstrating the usefulness of iLst996 as a broad GSM for *Lipomyces* metabolism. Pathways that diverged from iLst996 mainly revolved around alternate carbon metabolism, with ortholog groups excluding NRRL Y-11557 annotated to be involved in transport, glycerolipid, and starch metabolism, among others. Overall, this study provides a useful modeling tool and data for analyzing and understanding *Lipomyces* species metabolism and will assist further engineering efforts in *Lipomyces*.

## 1 Introduction

The *Lipomyces* clade consists of soil-dwelling oleaginous yeasts that possess a diverse set of saccharification enzymes and are known for their ability to accumulate triacylglycerol (TAG), a potential biofuel precursor. Due to their extensive number of carbohydrate-active enzymes (CAZymes), *Lipomyces* species have demonstrated growth on a wide range of carbon sources and waste feedstocks and have also displayed tolerance to inhibitory compounds found in hydrolysates (Deinema and Landheer, 1956; Angerbauer et al., 2008; Oguri et al., 2012; Xavier et al., 2017; Pomraning et al., 2019). Thus, *Lipomyces* have become attractive candidates for renewable biofuel and chemical production from waste feedstocks. The most well-studied species in the clade is *Lipomyces starkeyi*, which has been genome sequenced (Riley et al., 2016). Several *L. starkeyi* strains have been reported to accumulate over 60% of dry cell weight as TAG, with more than 80% lipid obtained (Azad, 2014; Zhou et al., 2021). The recent development of genetic engineering tools has further enabled the manipulation of lipid production properties (Calvey et al., 2014; Dai et al., 2017; McNeil and Stuart, 2018; Takaku et al., 2020); however, further improvement of lipid yields and productivities is needed to de-risk industrial production (Zhang et al., 2022).

Genome-scale metabolic models (GSMs) are tools that provide a convenient way to link genes to reactions (Han et al., 2023). Combining GSMs with flux balance analysis has proven to be an effective strategy for identifying engineering targets and optimal conditions for increased product yields and have been used to enhance biofuel production (Bro et al., 2006; Li et al., 2012; Xu et al., 2013). Oleaginous yeast-based GSMs have been used to gain an insight into lipid metabolism and identify optimal genetic engineering strategies for improved TAG production (Wei et al., 2017; Dinh et al., 2019; Ventorim et al., 2022). In *Yarrowia lipolytica*, a model oleaginous yeast, an insight into the lipid metabolism led to the identification of optimal production conditions that increased lipid yields (Kavscek et al., 2015).

A small-scale metabolic model was previously developed for *L. starkeyi* and used to model several strategies for improving lipid yields (Zhou et al., 2021). A more comprehensive GSM can provide further understanding of metabolism and allows for comparisons of reactions with GSMs of other species. Additionally, GSMs facilitate the use of computational algorithms to identify non-intuitive metabolic targets outside of central metabolism (Kim et al., 2019; McNaughton et al., 2021). Here, we develop a *L. starkeyi* GSM (iLst996) based on the genome of strain NRRL Y-11557 (Riley et al., 2016). We utilize omics data from a derived strain that produces less exo-polysaccharides (NRRL Y-11558 (Dai et al., 2019)) than the parent strain to correct the biomass equation and further validate the model on collected phenotypic data. We then sequence 25 other *Lipomyces* strains and examine the applicability of the built model for modeling the *Lipomyces* clade.

## 2 Materials and methods

### 2.1 Genome sequencing

For *L. starkeyi* CBS 7536, *L. tetrasporus* Phaff 51-55, *L. kononenkoae* CBS 7786, *L. doorenjongi* CBS 8726, *L. starkeyi* CBS 7851, *L. doorenjongii* NRRL Y-27504, *L. starkeyi* CBS 8064, *L. mesembrius* CBS 7600, *L. chichibuensis* CBS 12929, *L. japonicus* CBS 7319, *L. oligophaga* CBS 7107, *L. arxii* Phaff 12-163, and *Dipodascopsis tothii* CBS 759.85, a plate-based DNA library for Illumina sequencing was prepared on the Hamilton VANTAGE robotic liquid handling system using a Kapa Biosystems HyperPrep library preparation kit (Roche). A measure of 200 ng of genomic sample DNA was sheared to 600 bp using a Covaris LE220 Focused-ultrasonicator. The sheared DNA fragments were size-selected by double-SPRI using TotalPure NGS beads (Omega Bio-tek), and then, the selected fragments were end-repaired, A-tailed, and ligated with Illumina compatible unique dual-index sequencing adaptors (IDT, Inc.). The prepared libraries were then quantified using Kapa Illumina library quantification kits (Roche) and run on a LightCycler 480 real-time PCR instrument (Roche). The quantified libraries were multiplexed, and the pool of libraries was then prepared for sequencing on the Illumina NovaSeq 6000 sequencing platform using NovaSeq XP v1.5 reagent kits (Illumina) and an S4 flow cell, following a 2 × 150 indexed run recipe. For *L. tetrasporus* NRRL Y-11562, *L. tetrasporus* NRRL Y-8875, *L. orientalis* CBS 10300, *L. starkeyi* Phaff 55-103, *L. starkeyi* Phaff 78-25, *L. starkeyi* Phaff 78-24, *L. doorenjongii* Phaff 78-26, *L. kononenkoae* NRRL Y-11553, *Kockiozyma suomiensis* NRRL Y-17356, *Limtongia smithiae* NRRL Y-17922, and *Dipodascopsis uninucleata* Phaff 50-6, a plate-based DNA library for Illumina sequencing was prepared on the PerkinElmer Sciclone NGS robotic liquid handling system following the same protocol described above.

Following sequencing, all raw Illumina sequence data were filtered for artifact/process contamination using the JGI QC pipeline, and then, an assembly of the target genome was generated using a 20.00-M read-pair subsample of the resulting nonorganelle reads using SPAdes v3.15.2 (Bankevich et al., 2012). Prior to assembly, organellar contamination was removed using GetOrganelle.py (Jin et al., 2020).

For *Myxozyma melibiosi*, 2 µg of genomic DNA was treated with DNA prep to remove single-stranded ends and repair DNA damages, followed by end repair, A-tailing, and ligation with PacBio adapters using the SMRTbell Template Prep Kit 1.0 (Pacific Biosciences). The final size was selected using the Sage BluePippin system using a 6-kb lower cutoff. The PacBio sequencing primer was then annealed to the SMRTbell template library, and version P6 sequencing polymerase was bound to them. The prepared SMRTbell template libraries were then sequenced on a Pacific Biosciences RSII sequencer using version C4 chemistry and 1 × 240 sequencing movie run times. Filtered subread data were then assembled using Falcon version 0.7.3 to generate an initial assembly (Chin et al., 2016). The mitochondria data were assembled separately from the Falcon pre-assembled reads (preads) using an in-house tool (assemblemito.sh) to filter the preads and polished using Quiver version smrtanalysis_2.3.0.140936.p5 (https://github.com/PacificBiosciences/GenomicConsensus) (Chin et al., 2013). A secondary Falcon assembly was generated using the mitochondrion-filtered preads, improved using FinisherSC version 2.0 (Lam et al., 2015), and polished using Quiver version smrtanalysis_2.3.0.140936.p5 (https://github.com/PacificBiosciences/GenomicConsensus) (Chin et al., 2013). Statistics were based on 1 N to denote a gap. Contigs less than 1,000 bp were excluded.

### 2.2 Transcriptome sequencing and genome annotation

For all lineages except *M*. *melibiosi*, plate-based RNA sample prep was performed on the PerkinElmer Sciclone NGS robotic liquid handling system using an Illumina TruSeq Stranded mRNA HT sample prep kit, with the poly-A selection of mRNA as outlined by the Illumina user guide protocol: https://support.illumina.com/sequencing/sequencing_kits/truseq-stranded-mrna.html. The following conditions were used: the total RNA starting material was 1 µg per sample, and 8 PCR cycles were used for library amplification. The prepared libraries were quantified using the Kapa Illumina library quantification kit (Roche) and run on a LightCycler 480 real-time PCR instrument (Roche). The quantified libraries were then multiplexed, and the pool of libraries was then prepared for sequencing on the Illumina NovaSeq 6000 sequencing platform using NovaSeq XP v1.5 reagent kits (Illumina) and an S4 flow cell, following a 2 × 150 indexed run recipe. For *M. melibiosi*, stranded cDNA libraries were generated using the Illumina TruSeq Stranded RNA LT kit. mRNA was purified from 100 ng of total RNA using magnetic beads containing poly-T oligos. mRNA was fragmented using divalent cations at high temperatures. The fragmented RNA was reverse-transcribed using random hexamers and SSII (Invitrogen), followed by second-strand synthesis. The fragmented cDNA was treated with an end-pair, A-tailing, adapter ligation, and 10 cycles of PCR. The prepared library was quantified using Kapa Biosystems next-generation sequencing library qPCR kit (Roche) and run on a Roche LightCycler 480 real-time PCR instrument. The quantified library was then multiplexed with the other prepared libraries, with the pool of libraries prepared next for the Illumina HiSeq sequencing platform via a TruSeq paired-end cluster kit v4 and an Illumina cBot instrument to generate a clustered flow cell for sequencing. The flow cell was sequenced on the Illumina HiSeq 2500 sequencer using HiSeq TruSeq SBS sequencing kits, v4, following a 2 × 150 indexed run recipe.

Following sequencing, raw FASTQ file reads were filtered and trimmed using the JGI QC pipeline. Using BBDuk (BBTools version 38.79), the raw reads were evaluated for artifact sequences by k-mer matching (k-mer = 25), allowing 1 mismatch. The detected artifacts were trimmed from the 3′end of the reads. RNA spike-in reads, PhiX reads, and reads containing any Ns were removed. Quality trimming was performed using the Phred trimming method set at Q6. Following trimming, reads under the length threshold were removed (minimum length 25 bases or 1/3 of the original read length—whichever is longer). The filtered FASTQ files were used as the input for the *de novo* assembly of RNA contigs. Reads were assembled into consensus sequences using Trinity v2.11.0 (v2.3.2 for *M. melibiosi*) (Grabherr et al., 2011). Following genome and transcriptome sequencing, all genomes were annotated using the JGI annotation pipeline (Grigoriev et al., 2013). CAZyme annotations were obtained after a semi-manual curation of protein-filtered model sequences by the Carbohydrate-Active enZYmes (CAZy) team (www.cazy.org (Drula et al., 2021)).

### 2.3 Genome-scale model construction and validation

**FIGURE 1:**
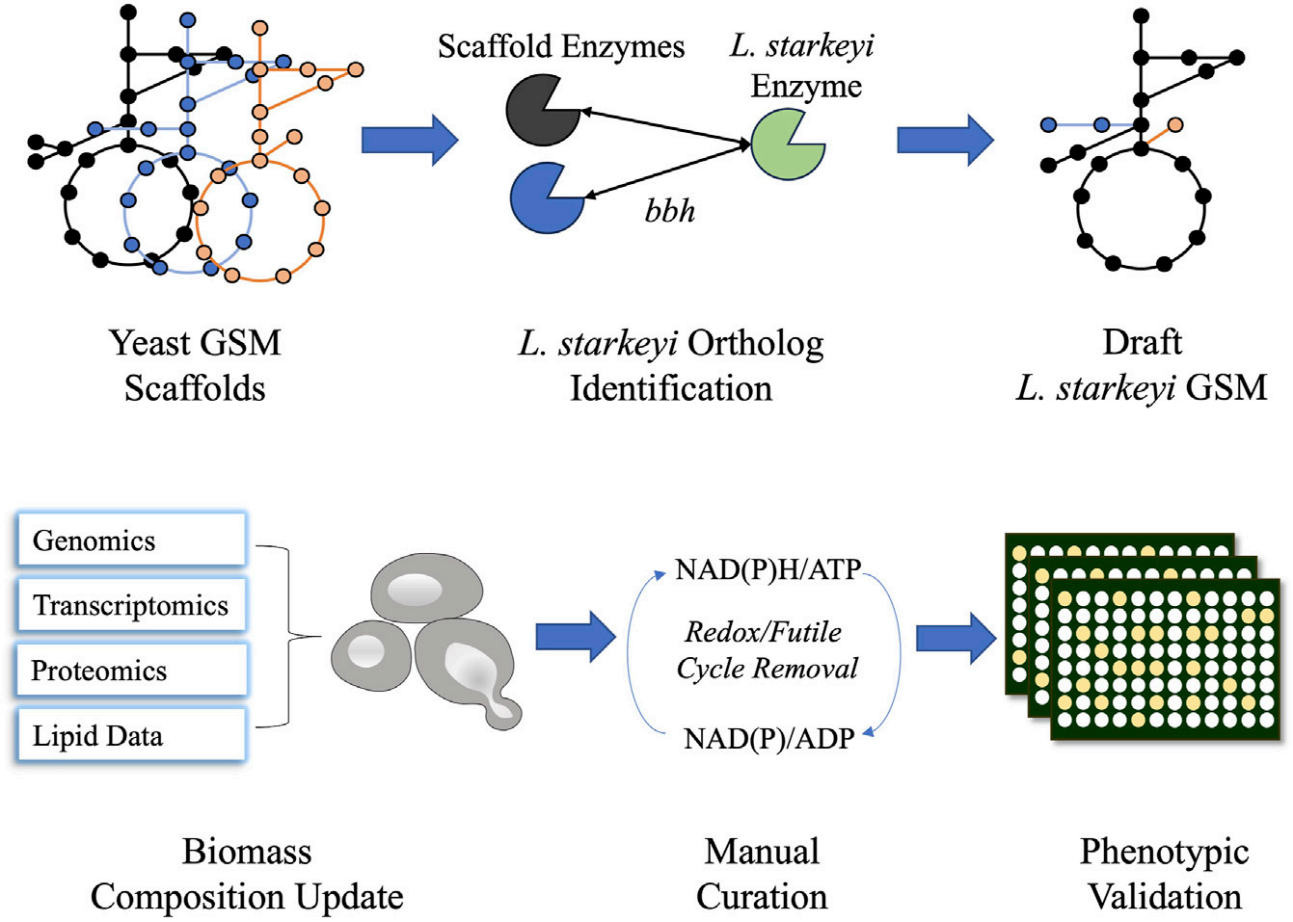
Schematic diagram of iLst996 genome-scale metabolic model (GSM) construction. *Lipomyces starkeyi* orthologs were identified using the NCBI blast tool for bidirectional best hits (bbhs) and the OrthoMCL pipeline. Orthologs were identified in three other yeast GSMs and were used to develop the initial draft GSM. The biomass composition and lipid synthesis reactions were then updated using *L. starkeyi* omics and lipid data. Manual curation was performed to remove futile cycles, leading to realistic flux predictions. Phenotypic microarray plates were then used to assess the accuracy of the model.

The NRRL Y-11557 genome sequence was published in a previous study (Riley et al., 2016). The OrthoMCL pipeline was employed to identify *L. starkeyi* orthologs in the *Rhodosporidium toruloides* IFO0880, *Y. lipolytica* CLIB122, and *Saccharomyces cerevisiae* S288C genomes (Li et al., 2003). The respective GSMs of the species, Rt_IFO0880 (Kim et al., 2020), iYLI647 (originally published by Mishra et al. (2018) and updated by Xu et al. (2020)), and Yeast8 (Lu et al., 2019), were utilized to construct *L. starkeyi* GSM iLst996. Reactions corresponding to genes with *L. starkeyi* orthologs were imported to the iLst996 model, and the reaction gene associations were updated to reflect the *L. starkeyi* genes (see Supplementary Material S1 and https://github.com/AgileBioFoundry/LstarkeyiGSM). IFO0880 was used as the initial scaffold, with *S. cerevisiae* and *Y. lipolytica* models used to identify potential gaps or missed annotations. The reaction naming convention was adjusted to follow the BiGG GSM format (King et al., 2016). All gene reaction rules were set to “OR” in the GSM. Biomass composition was updated using transcriptomic and proteomic data collected from batch bioreactor growths on glucose and xylose containing a minimal medium (https://github.com/AgileBioFoundry/LstarkeyiGSM) and previously published lipid (Calvey et al., 2016) species and composition data (Itoh and Kaneko, 1974; Suzuki and Hasegawa, 1974; Kaneko et al., 1976) using the Python package BOFdat (Lachance et al., 2019). The lipid data were also used to update lipid molecular species and lipid synthesis reactions in the model. The utilized published fatty acid compositional data were from strain NRRL Y-11557 grown under five different conditions, while the lipid macromolecule data were collected from *L. starkeyi* strain IAM-4753 ((Itoh and Kaneko, 1974). Gap filling was performed using the COBRApy gap-fill function to enable growth (Ebrahim et al., 2013). Futile cycles and missing reactions were identified using parsimonious flux balance analysis. Reactions involved in redox cycles had bounds modified to prevent the redox cycle. The reactions that were involved in the blocked futile cycles are two alcohol dehydrogenases, ALCD2y and ALDD19x_P, and a homoserine dehydrogenase HSDy. The blocked reactions are further documented in https://github.com/AgileBioFoundry/LstarkeyiGSM. Non-growth-associated maintenance (NGAM) was experimentally determined for growth on xylose (Anschau and Franco, 2015a) and was used as the NGAM value in the GSM. Growth-associated maintenance (GAM) was estimated using published continuous cultivation data during growth on glucose and xylose (Anschau and Franco, 2015b). DeepLoc v 2.0 and BUSCA were utilized to predict protein subcellular locations (Almagro Armenteros et al., 2017; Savojardo et al., 2018). The metabolic flux distribution was modeled using COBRApy (Ebrahim et al., 2013). Model quality evaluation was analyzed via MEMOTE (Lieven et al., 2020). The iLst996 GSM construction process was documented in Jupyter Notebooks (https://github.com/AgileBioFoundry/LstarkeyiGSM), and an overview is given in Figure 1. The respiratory quotient for each GSM was determined using the COBRApy “model.summary” function during growth on 1 mmol/gDCW/h of glucose. The quotient was calculated by dividing the absolute value of the carbon dioxide flux released (HCO_3_^−^ was added to the carbon dioxide released for models in which it was secreted) by the oxygen uptake. In our hands, we observed glucose uptake rates reach as high as 1.5 mmol/g DCW/h, with a corresponding growth rate of ∼0.07–0.1/h. We set the model uptake to 1 mmol/g DCW/h as an easy-to-use realistic approximation.

### 2.4 Phenotypic data collection

Biolog Inc. (Hayward, CA) phenotypic microarray plates were used to collect growth/no-growth data on metabolic substrates. Biolog 96-well plates PM1, PM2A, PM3B, and PM4A were used for the phenotypic assays, along with Biolog Dye D. Each well contains a different carbon (plates PM1 and PM2A), nitrogen (PM3B), phosphate, or sulfur source (both in PM4A) for growth evaluation. *L. starkeyi* strain NRRL Y-11558 (a mutant of NRRL Y-11557, which produces less exo-polysaccharides (Dai et al., 2019)) was grown on YPD plates at 28°C for 4–5 days. The strain was then scraped, resuspended in sterile water, and used to inoculate Biolog inoculation fluid (IFY-0) to an OD_600_ value of 0.005. For nitrogen (PM3B), phosphorus, and sulfur sources (PM4A), 100 mM of glucose was added to IFY-0. Microarray plates were inoculated with 100 μL of cell suspension, cultured at 28°C, and shaken at 800 rpm on a microplate shaker (Fisherbrand, Waltham, MA). Growth and metabolic activity were determined via measuring the reduction in the dye at 750 nm.

### 2.5 Media and growth conditions for genomic sequencing

Strains were cultivated on a YPD medium (10 g/L yeast extract, 10 g/L peptone, and 20 g/L glucose) in shake flasks at 28°C at 200 rpm in an incubated orbital shaker to produce biomass. Genomic DNA was extracted by the CTAB method, as previously described (Dai et al., 2021), and RNA was extracted from samples using a Maxwell 16 LEV Plant RNA kit (Promega, Madison, WI). The samples were sequenced on an Illumina platform and annotated by the Joint Genome Institute as described in Method Sections 2.1 and 2.2 and are available on the MycoCosm portal (https://mycocosm.jgi.doe.gov/mycocosm/home (Grigoriev et al., 2013)).

### 2.6 Evaluating iLst996 model applicability

The JGI-generated protein files for each of the sequenced *Lipomyces* species, along with *Y. lipolytica* FKP355 (Pomraning et al., 2018) and *Babjeviella inositovora* NRRL Y-12698 (Riley et al., 2016), were used in the OrthoMCL pipeline (Li et al., 2003). Ortholog groups (OGs) were obtained from the pipeline and used to assess the relatability of species and gene inclusion. The *L. starkeyi* NRRL Y-11557 JGI-generated Kyoto Encyclopedia of Genes and Genomes (KEGG) and Eukaryotic Orthologous Groups (KOGs) of protein annotations were used to label each OG when determining the conservation of metabolism. The presences or absence of orthologs for each gene in the iLst996 model was determined for individual species through the examination of OG members for the specified iLst996 gene. Reaction inclusion was determined by ensuring that at least one gene for the specified reaction was in the particular species. Annotations from JGI-generated KOG or KEGG terms for each species were combined when generating consensus annotations for each OG when an NRRL Y-11557 gene was not present.

### 2.7 Heatmaps, dendrogram generation, and visualization

The Biolog heatmaps were generated using the following procedure: a threshold was chosen based on the values from each substrate and plate negative control (i.e., carbon, nitrogen, phosphate, and sulfur). The maximum values reached were then recorded, and the span was split into four steps (based on the threshold and maximum value). An integer (1–4) was used to describe the growth on each substrate, with 0 being assigned to wells below the threshold. For dynamic data, the maximum integer reached was used to describe the growth. The Seaborn package was used to generate the heatmap via the “seaborn.heatmap” function (Waskom, 2020). The dendrogram was generated according to the following procedure: a co-occurrence matrix of species genes was constructed for each OG. Multiple genes from a species were considered as one occurrence in an OG. A cosine similarity matrix was then constructed using scikit-learn (Pedregosa et al., 2011) and SciPy library (Virtanen et al., 2020) functions “pairwise_distance” and “cosine,” respectively. The Seaborn (Waskom, 2020) and Matplotlib (Hunter, 2007) python packages were utilized for visualization.

## 3 Results

### 3.1 Genome-scale model construction

The published genome of *L. starkeyi* strain NRRL-11557 was utilized to construct *L. starkeyi* GSM iLst996. The construction procces was documented in Jupyter Notebooks (https://github.com/AgileBioFoundry/LstarkeyiGSM), and an overview is provided in Figure 1. Three high-quality published yeast GSMs were utilized as scaffolds for iLst996 construction: *Y. lipolytica* (iYLI647), *S. cerevisiae* (Yeast8), and *R. toruloides* (Rt_IFO0880). The scaffolds were chosen based on the extensive annotation of metabolism for their respective species and because models Rt_IFO0880 and iYLI647 represent oleaginous yeast metabolic networks. Metabolic reactions were directly imported to iLst996 if there was an *L. starkeyi* ortholog for the corresponding scaffold gene/genes. The biomass composition, lipid bodies, and lipid synthesis reactions were updated using experimental data as described in Methods Section 2.1 *Genome-scale model construction and validation*. We also updated the non-growth-associated ATP maintenance (NGAM) and growth-associated ATP maintenance (GAM) demands to reflect *L. starkeyi*. GAM was estimated from published experimental growth data under continuous cultivations, while NGAM was previously experimentally determined under xylose growth conditions (Anschau and Franco, 2015b). Interestingly, the NGAM value was much lower than what was predicted for *R. toruloides,* while the GAM requirement was slightly lower (111 mmol/gDCW/h vs. 105 mmol/gDCW/h for GAM, 1.2 vs. 0.4 mmol/gDCW/h for NGAM). The updated biomass equation is given in Supplementary Table S1. The final model *L. starkeyi* GSM iLst996 contained 2,193 reactions, 1,909 metabolites, and 996 genes. The model was then verified through MEMOTE, a platform used to determine the quality of metabolic models (Lieven et al., 2020). iLst996 had a MEMOTE score of 83%, with high scores in annotations and consistency.

### 3.2 *L. starkeyi* NRRL Y-11557 phenotypic growth and iLst996 validation

**FIGURE 2:**
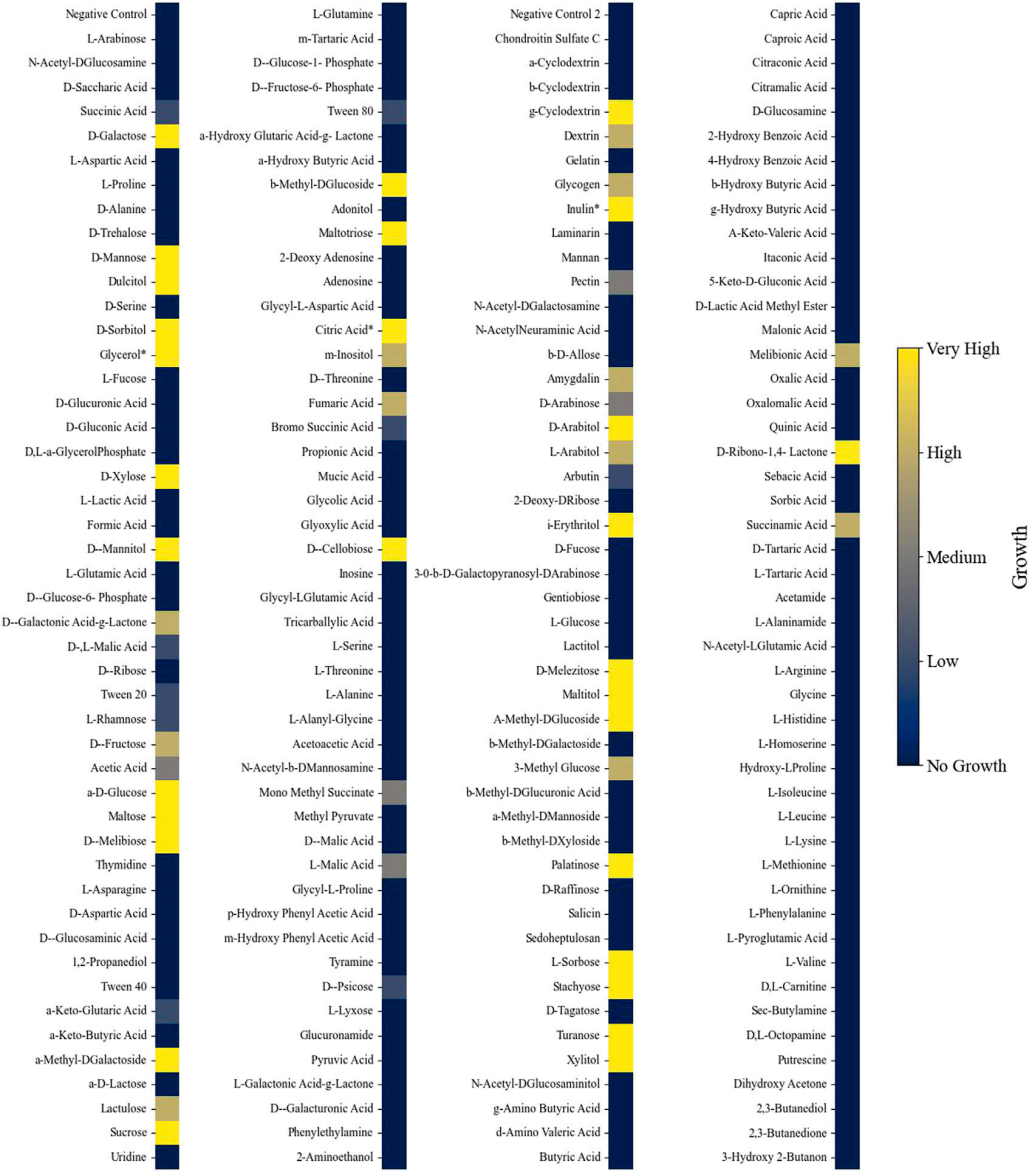
Heatmap indicating growth of *L. starkeyi* on different carbon substrates. Asterisks (*) indicate values that were corrected based on the literature evidence. Growth was classified into categories based on the maximum OD_750_ value achieved on each substrate compared to the negative control for each nutrient source and plate.

Phenotypic growth data from different nutrient sources were collected from Biolog phenotypic microarrays plates (PM1, PM2A, PM3B, and PM4A). Each 96-well plate contains a different carbon (plates PM1 and PM2A), nitrogen (PM3B), phosphate, or sulfur (PM4A) source in each well for growth evaluation. The substrates cover a broad range of chemicals, such as organic acids, saccharides, and amino acids. A dye is also added to each well, and when the dye becomes reduced during growth, there is a change in color. Growth was observed in 54% of the Biolog nutrient conditions (206 out of 379 (excluding the negative controls); Supplementary Figures S1–S10). In three instances, the phenotypic assay results contradicted those observed in the literature and were corrected to indicate growth (Naganuma et al., 1985; Liu et al., 2017). *L. starkeyi* showed an extensive ability to grow on carbohydrate substrates but displayed limited growth on organic acid compounds (Figure 2). Of the different substances tested, 213 had corresponding metabolites in the GSM, with 123 of those nutrients supporting growth. Initially, the GSM was unable to predict growth under many sugar phosphate conditions that experimentally demonstrated growth. Gap filling indicated that reactions catalyzed by secreted phosphatases can enable growth. Several phosphatases were predicted to be extracellular in *L. starkeyi* NRRL Y-11557, and thus, the reactions were included in the GSM (localization predicted via DeepLoc version 2 and BUSCA (Almagro Armenteros et al., 2017; Savojardo et al., 2018)). However, the sugar phosphate catabolism in *L. starkeyi* likely needs further investigation to confirm mechanisms and pathways. Overall, the final model (iLst996) correctly predicted growth in 89 of the 123 growing conditions and had a total accuracy of 72% (34 conditions were incorrectly predicted to grow).

**FIGURE 3:**
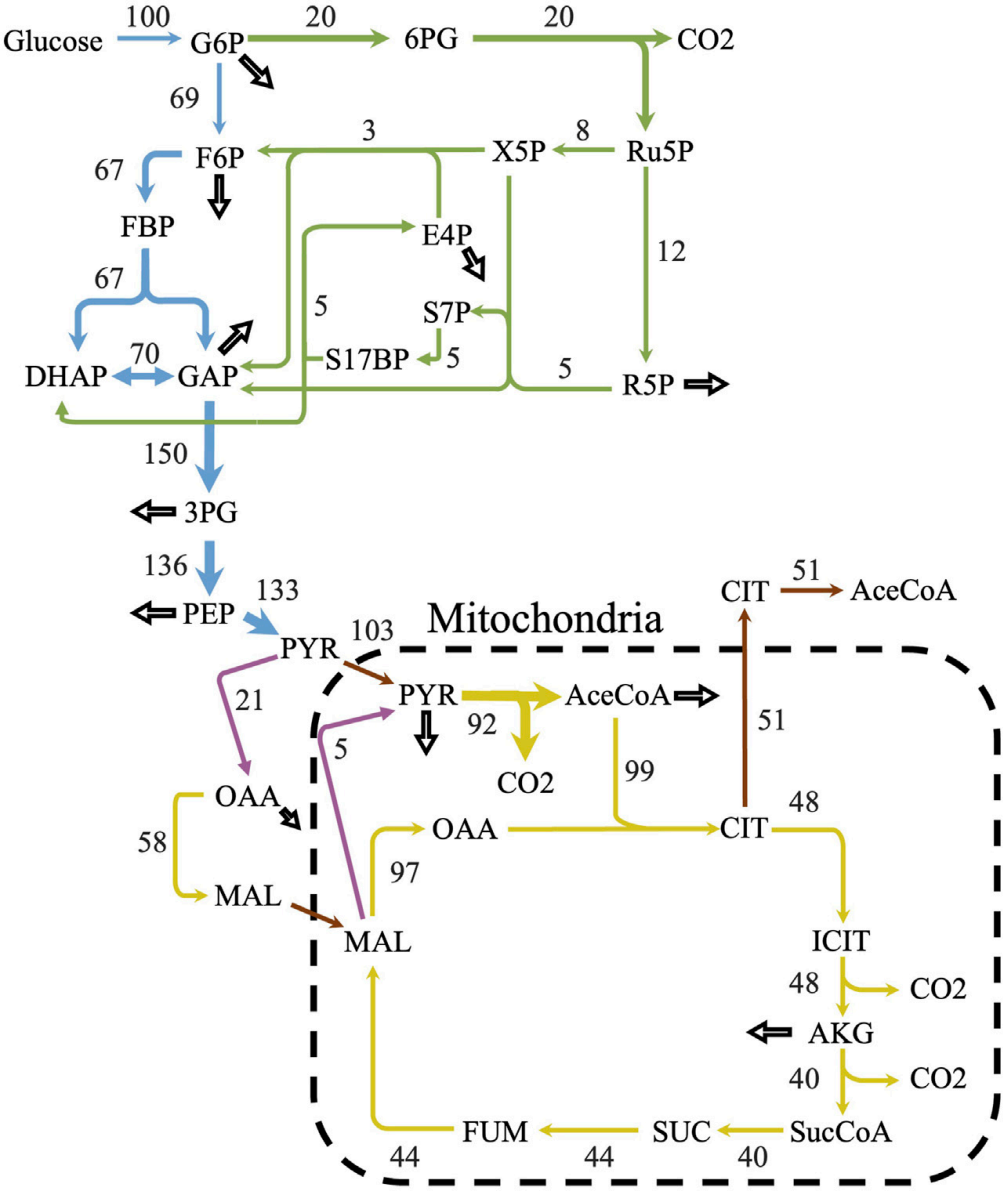
Predicted flux distribution of central carbon metabolism. The central carbon predicted flux values using parsimonious flux balance analysis and with an uptake of 1 mmol/gDCW/h of glucose. Flux values were normalized to a percentage of the carbon source uptake. Colors correspond to different metabolic pathways. Blue—glycolysis; green—pentose phosphate pathway; yellow—citric acid cycle; purple—anaplerotic reactions; and brown—transport reactions. Hollow arrows represent biomass drainage reactions. Some arrows represent lumped reactions for ease of visualization.

iLst996 predicted a growth rate of 0.089/h with 1 mmol/gDCW/h of glucose as a carbon source, the second highest of the scaffolded models (Table 1). The predicted growth rate was consistent with our experimental observations, where the growth rate ranged from 0.07 to 0.1/h and observed glucose uptake rates reached as high as 1.5 mmol/g/DCW/h. Interestingly, pFBA of iLst996 also predicted a lower oxygen demand (lower CO_2_ release than *R. toruloides* and *S. cerevisiae*) and, therefore, a higher respiratory quotient than the other scaffold models (Table 1). Examining the pFBA-predicted flux network indicated that 20% of the uptaken glucose carbon is routed through the pentose phosphate pathway (PPP; Figure 3), with approximately 70% of the glucose carbon entering glycolysis. The remaining carbon was directed toward glucose-1-phosphate, a precursor for cell wall components. The citrate synthesis step was predicted to be highly active, although nearly half of the flux entering the step was exported to the cytoplasm for acetyl-CoA (AceCoA) synthesis through ATP citrate lyase. The remaining flux proceeded through the tricarboxylic acid cycle (TCA). Anaplerotic reactions were moderately active (∼10–20% of the glucose carbon uptake flux).

**TABLE 1:** Summary of scaffold models and iLst996 growth, CO_2_ generation, and O_2_ uptake on 1 mmol/gDCW/h of glucose.

	*Saccharomyces cerevisiae*	*Rhodosporidium toruloides*	*Yarrowia lipolytica*	*Lipomyces starkeyi*
**mu (h** ^**-1**^ **)**	0.082	0.073	0.114	0.089
**CO**_**2**_ **secretion** $\frac{mmol}{gDCW.h}$	2.468	2.561	1.679	2.253
**O2 uptake** $\frac{mmol}{gDCW.h}$	2.332	2.287	1.499	1.799
**HCO**_**3**_^**−**^ **secretion** $\frac{mmol}{gDCW.h}$	-	0.002	-	0.002
**Respiratory quotient**	1.058	1.121	1.120	1.253

Further examination of the cofactor and energy balances revealed three redox cycles involving NADPH and NADH. Two of the cycles were involved in acetaldehyde/ethanol metabolism (reaction IDs: ALCDy2/ALCD2x and ALDD19x_P/ALDD19xr), while the third cycle was involved in a homoserine dehydrogenase reaction (reaction IDs: HSDy/HSDxi). Blocking the redox cycles (see *Materials and Methods* and Supplementary Material S1) resulted in a predicted flux shift, with nearly 70% of the glucose uptake flux being routed through the PPP and an increase in anaplerotic reaction activity (Supplementary Figure S11). GSM redox reactions can be difficult to constrain without ^13^C-metabolic flux analysis (MFA) data. Although data were not available for *L. starkeyi*, ^13^C-MFA was performed in closely related oleaginous organisms, including the yeast *Y. lipolytica* and the fungus *Mucor circinelloides*. Both organisms had relatively high PPP flux, with 30%–52% of the uptaken glucose routed through the pathway, consistent with the predicted *L. starkeyi* flux (Christen and Sauer, 2011; Wasylenko et al., 2015; Zhao et al., 2015).

### 3.3 Growth characteristics, lipid yields, and gene essentiality

We next utilized the model to determine lipid yields from diverse carbon sources (Supplementary Table S2). A small-scale *L. starkeyi* metabolic reconstruction that contained approximately 130 reactions had previously been used to assess lipid yields (Zhou et al., 2021). Small-scale models can often represent core metabolism with a good degree of accuracy, and comparing theoretical lipid yields from the small-scale model with iLst996 indicated that the results were consistent (using the blocked futile cycle model). iLst996 did predict higher theoretical yields than the smaller model, which may be due to potentially more NADPH generation routes in the genome-sized model, and indicates that there are differences in metabolism that can influence final yields that were not captured in smaller-scale models.

Another useful feature of GSMs is their ability to *in silico* predict gene essentiality by removing genes and associated reactions from the model and assessing if biomass can still be formed. iLst996 predicted 202 genes to be essential, with the corresponding reactions spread across metabolism (Supplementary Figure S12 and Supplementary Material S2). Many of the predicted essential genes were involved in cofactor, amino acid, and nucleotide biosyntheses.

### 3.4 *Lipomyces* clade sequencing and iLst996 applicability

**FIGURE 4:**
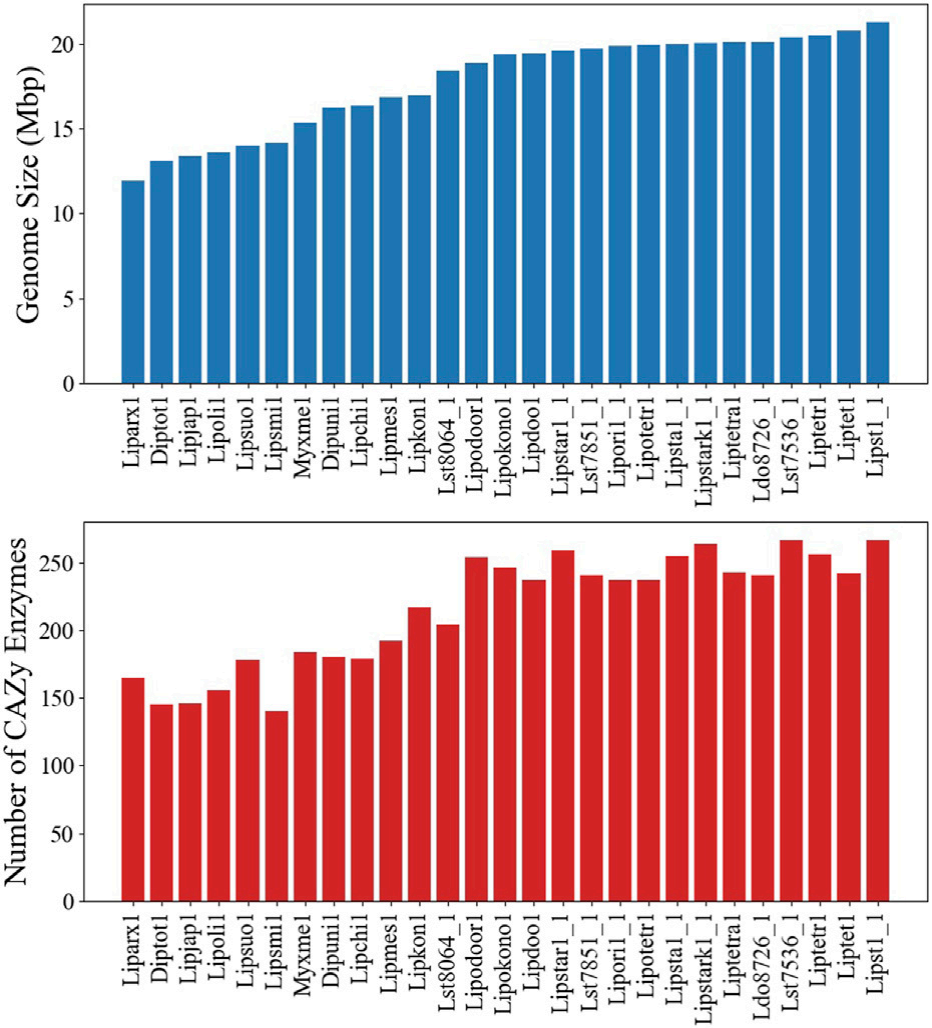
Genome and carbohydrate-active enzyme (CAZyme) characteristics of the *Lipomyces* clade. **(Top)** The genome sizes of the species. **(Bottom)** The number of CAZymes annotated in each genome.

Although a GSM, based on a single strain, is an invaluable tool, extendibility to other strains and species can be limited. To assess the model usability in the *Lipomyces* clade, we sequenced 25 other *Lipomyces* members and analyzed the similarity of metabolism. The published genome of *L. tetrasporus* NRRL Y-64009 was also included in the analysis (Jagtap et al., 2023). The full lists of species and an overview of their genome characteristics are provided in Supplementary Material S3. *L. starkeyi* NRRL Y-11557 had the largest assembled genome (21.3 Mbp) and the highest number of predicted genes (8,192; Supplementary Figure S13). The majority (60%) of the sequenced strains had genome sizes >18.4 Mbp and >7,000 proteins. There were 6 species with genome sizes <14.2 Mbp, with *L. arxii* Phaff 12-163 having the smallest genome at 11.9 Mbp (Figure 4).

**FIGURE 5:**
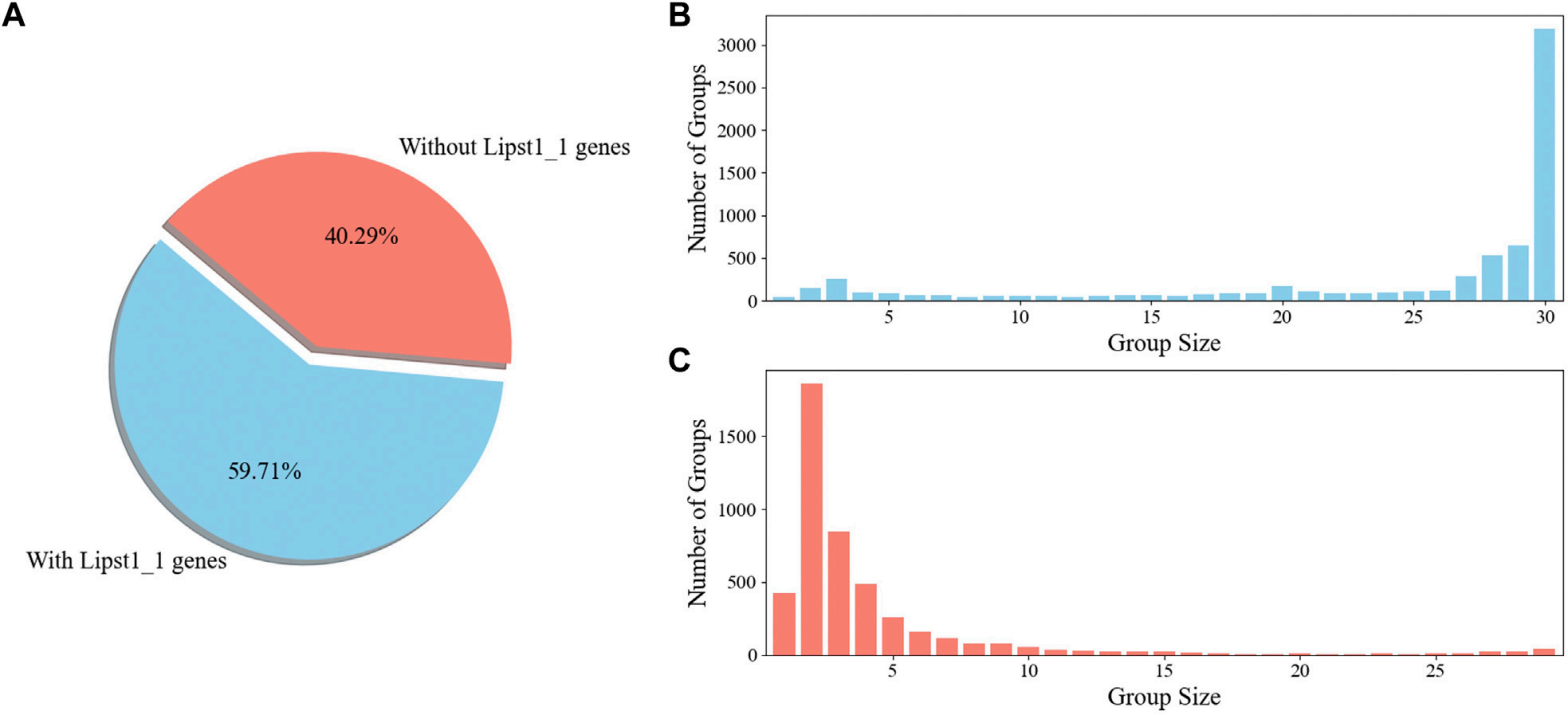
OrthoMCL ortholog group (KOG) breakdown **(A)**. Percentage of OGs with and without a gene in the *L. starkeyi* NRRL Y-11557 strain (Lipst1_1) used to build GSM iLst996 **(B)**. Sizes of the OGs containing *L. starkeyi* NRRL Y-11557 genes **(C)**. Sizes of OGs without *L. starkeyi* NRRL Y-11557 genes.

To gain an insight into the applicability of iLst996 to the *Pan* species clade, an OrthoMCL pipeline was ran with the 25 newly sequenced strains and *L. starkeyi* NRRL Y-11557. *Y. lipolytica* FKP355 (Madzak et al., 2000) and *B. inositovora* NRRL Y-12698 (Riley et al., 2016) were included in the analysis as two outgroup species. Nearly 60% of the obtained OGs contained at least one gene from our base strain (NRRL Y-11557, Figure 5). The newly sequenced species had at least one predicted protein in over 80% of the groups, with NRRL Y-11557 genes (Supplementary Figure S14). OGs that did not contain an NRRL Y-11557 gene had a small number of proteins, indicating relatively small differences between species.

**FIGURE 6:**
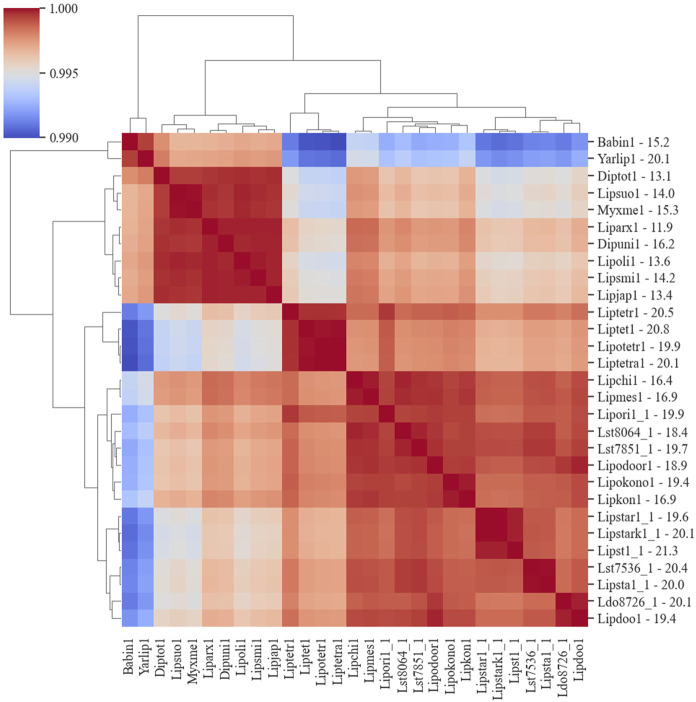
Phylogenic diagram of species based on the co-occurrence of proteins in OGs. The dendrogram was constructed from data based on the co-occurrence of species genes in the ortholog groups. The numbers on the *y*-axis after the JGI identification indicate the size of the genome in Mbp.

The co-occurrence of species genes in an OG was generated from the OrthoMCL results and was used to construct a similarity matrix and a phylogenetic tree (Figure 6). As expected, *Y. lipolytica* and *B. inositovora* were the most phylogenetically distant species. *L. starkeyi* strains and more closely related species formed a distinct group from the *L. tetrasporus* strains. The strains with smaller genomes were grouped together and were also more evolutionary distant to the *L. tetrasporus* and *L. starkeyi* strains. *L. starkeyi* is notable for its expanded repertoire of CAZymes and ability to consume a wide variety of carbohydrates compared to most *Saccharomycotina* yeasts (Riley et al., 2016). The strains with larger genome sizes were also correlated with the number of CAZymes predicted to be within each group (Figure 4). Glycoside hydrolase family (GH2, GH3, GH13, GH25, GH17, GH18, GH27, GH32, and GH78), glycosyltransferase family (GT15, GT25, GT32, and GT34), and auxiliary activity family (AA6) genes are enriched in the genomes of *L. starkeyi* and closely related species in the genus (*L. doorenjongii*, *L. orientalis*, and *L. kononenkoae*), suggesting the expanded role these organisms play in the production and degradation of carbohydrates and lignin breakdown products in the environment. Of the 243 annotated CAZymes in *L. starkeyi* NRRL Y-11557, 69 were included in iLst996 (Supplementary Material S3).

Finally, to determine the applicability of iLst996 to the clade, the presence or absence of an OG for each reaction in the GSM was determined for every species (Supplementary Material S4). In the context of the GSM, most reactions in central carbon metabolism were conserved in closely related species. Supplementary Figure S15 visualizes the percentage of conserved metabolic reactions for the reaction network. Individual species had various orthologs absent in the *L. starkeyi*-specified genes, which are provided in Supplementary Material S4. The largest deviation of conserved pathways occurred in alcohol and alternate carbon metabolism (e.g., butanoate metabolism). Drilling down into the ortholog groups that did not include *L. starkeyi* NRRL Y-11557 revealed that there were several proteins common across many of the species. Many of the unique proteins were involved in galactose, starch, and glycerolipid pathways, once again reflecting the diversity of the saccharification enzymes found in the clad. On further examination, there were also 3-oxoacyl-ACP reductases and NADPH:quinone reductases in 15/26 of the species, as well as protochlorophyllide reductases annotated in 11 species (Supplementary Material S3).

## 4 Discussion

Oleaginous microbes require a large supply of reducing equivalents to support the generation of lipids. Often, the reducing requirement is met through NADPH generation via the oxidative portion of PPP activity, especially in yeasts that lack a NADPH-dependent malic enzyme, like *L. starkeyi* and *Y. lipolytica*. The iLst996 genome-scale model predicted that approximately 20% of the consumed glucose is shunted through the oxidative portion of the PPP during growth. However, this percentage was predicted with the activity of three identified redox cycles (involving reactions ALCDy2/ALCD2x and ALDD19x_P/ALDD19xr and HSDy/HSDxi). Blocking the cycles (see *Materials and Methods* and Supplementary Material S1) led to a high portion of flux shifting to the PPP (71%), which met nearly 90% of the *L. starkeyi* NADPH requirement. While seemingly high, ^13^C-MFA experimentally assesses that *Y. lipolytica* sends approximately 40%–50% of its glucose uptake flux through the PPP (Christen and Sauer, 2011; Wasylenko et al., 2015) and that two strains of the oleaginous fungus *M. circinelloides* have similar PPP flux ranges (∼30–50%) (Zhao et al., 2015). Yeasts lack a nucleotide transhydrogenase for interconversion of NADPH and NADH that is often present in bacteria (Nissen et al., 2001) and may rely on other reaction mechanisms to balance redox states. Thus, iLst996 is consistent with experimental observations in other oleaginous fungi.

Further research has demonstrated that flux through the PPP is correlated with biomass yields from glucose, with Crabtree-positive yeasts that produce fermentation products having a lower PPP flux than Crabtree-negative yeasts (Blank et al., 2005). Preventing ethanol formation in *S. cerevisiae* may have led to 90% of the carbon uptake flux being diverted to the PPP (Jessop-Fabre et al., 2019), although the authors noted experimental discrepancies in other studies. *L. starkeyi* produces relatively limited amounts of byproducts and has higher lipid yields than *Y. lipolytica*, which often secretes high amounts of organic acids (Erian et al., 2020). Thus, the higher yield likely requires more NADPH per generation, which would be consistent with the higher PPP activity predicted in *L. starkeyi* when the redox cycles are blocked.

Phylogenetic trees are typically constructed using conserved sequences of the 16S ribosomal subunit. Prior work in the *Lipomyces* clade examined the phylogenic tree of many of the species explored here (Bruce et al., 2016). Using OGs complements the 16S phylogenic tree analysis and allowed a more nuanced view of protein presences and absences for the purpose of examining GSM applicability. Although this approach will miss paralogs or other enzymes that may perform a similar function, it allows researchers to identify potential gaps in the reactions of the other clade members. Thus, the data enable easier applicability of iLst996 to other *Lipomyces* species. This is of particular importance as some species, such as *L. tetrasporus*, have higher lipid yields than *L. starkeyi* (Bruce et al., 2016). Our reaction presence/absence data indicated that many of the main carbon pathways were present at 100% (Supplementary Material S5), with the largest deviations occurring in alternate carbon metabolism and sugar conversion. As soil-dwelling microorganisms, the *Lipomyces* clade may have faced more evolutionary pressure to adapt to specific carbon compounds in their local environment, leading to more distinct carbon pathways. Indeed, *Lipomyces* are known for having a large number of CAZymes, and the phenotypic analysis here demonstrated the ability of *L. starkeyi* to degrade a wide range of saccharide carbon sources. Of these CAZymes, nearly 28% were captured in iLst996. Thus, there is a significant gap between what the base genome model predicts for metabolism and the ability of *L. starkeyi* NRRL Y-11557 to degrade various carbohydrate compounds. Further work building the GSM pathways to account for more of the catabolic pathways would increase the predictive power of modeling *L. starkeyi*. Interestingly, more limited growth was observed on organic acids, such as succinic and acetic acid, with no growth observed on several other organic acids (Figure 2). The species utilized a diverse set of phosphate and sulfur carbon sources, which can have benefits for industrial uses, in which cheap nutrients can be used instead of more expensive sources.

Overall, the developed GSM is a useful tool that can be used in combination with computational strain design algorithms to identify strategies for engineering and improving production strains. New algorithmic methods such as Bayesian metabolic control analysis or the environmental version of minimization of metabolic adjustment (eMOMA) provide ways for combining GSMs and omics data for identifying non-intuitive targets for strain engineering (Kim et al., 2019; McNaughton et al., 2021). Furthermore, the model provides a more comprehensive map of *L. starkeyi* metabolism.

### 4.1 Limitations

One of the limitations of the construction of the GSM using a smaller set of orthologous organisms is that many of the genes remain experimentally unverified. As such, many of the gene–protein–reaction (GPR) rules were left only as “OR” rules (i.e., either gene A or gene B as opposed to gene A and gene B contribute to the reaction). Despite this limitation, the GSM GPR rules can provide a starting point for launching further investigation into genetic targets through the use of computational strain design algorithms (such as Bayesian metabolic control analysis, eMOMA, and OptKnock (Burgard et al., 2003; Kim et al., 2019; McNaughton et al., 2021)). Similarly, many of the predicted essential genes are unverified. As experimental evidence grows, the model will be continuously modified to reflect our understanding of *Lipomyces* metabolism and physiology. Further work generating fitness libraries will help with the curation and validation of the GSM.

## Data Availability

The genomes sequenced in our study were deposited in the NCBI database with the following accession numbers: *Lipomyces starkeyi* CBS 8064: PRJNA1069688; *Lipomyces doorenjongii* CBS 8726: PRJNA1069689; *Lipomyces tetrasporus* NRRL Y-11562: PRJNA1069690; *Lipomyces tetrasporus* NRRL Y-8875: PRJNA1069691; *Lipomyces doorenjongii* NRRL Y-27504: PRJNA1069692; *Lipomyces kononenkoae* NRRL Y-11553: PRJNA1069693; *Dipodascopsis tothii* CBS 759.85: PRJNA1069694; *Dipodascopsis uninucleata* Phaff 50-6: PRJNA1069695; *Lipomyces arxii* Phaff 12-163: PRJNA1069696; *Lipomyces chichibuensis* CBS 12929: PRJNA1069697; *Lipomyces japonicus* CBS 7319: PRJNA1069698; *Lipomyces mesembrius* CBS 7600: PRJNA1069699; *Lipomyces oligophaga* CBS 7107: PRJNA1069700; *Lipomyces orientalis* CBS 10300: PRJNA1069701; *Limtongia smithiae* NRRL Y-17922: PRJNA1069702; *Lipomyces tetrasporus* Phaff 51-55: PRJNA1069703; *Lipomyces starkeyi* Phaff 55-103: PRJNA1069704; *Lipomyces starkeyi* Phaff 78-24: PRJNA1069705; *Lipomyces starkeyi* Phaff 78-25: PRJNA1069706; *Lipomyces doorenjongii* Phaff 78-26: PRJNA1069707; *Lipomyces starkeyi* CBS 7536: PRJNA1069708; *Lipomyces kononenkoae* CBS 7786: PRJNA1069709; *Lipomyces starkeyi* CBS 7851: PRJNA1069710; *Kockiozyma suomiensis* NRRL Y-17356: PRJNA1071259; *Myxozyma melibiosi* Phaff 52-87: PRJNA368365. iLst996 was deposited in BioModels (Malik-Sheriff et al., 2020) and assigned the identifier MODEL2403190001. iLst996, code for reproducing the work, and data used to construct the model, including transcriptomic and proteomic datasets, are deposited at https://github.com/AgileBioFoundry/LstarkeyiGSM.

## Abbreviation

Lipst1_1, *Lipomyces starkeyi* NRRL Y-11557; Liptetr1, *Lipomyces tetrasporus* NRRL Y-11562; Liptet1, *Lipomyces tetrasporus* NRRL Y-64009; Liptetra1, *Lipomyces tetrasporus* NRRL Y-8875; Lst7536_1, *Lipomyces starkeyi* CBS 7536; Lipotetr1, *Lipomyces tetrasporus* Phaff 51–55; Lipokono1, *Lipomyces kononenkoae* CBS 7786; Ldo8726_1, *Lipomyces doorenjongii* CBS 8726; Lipori1_1, *Lipomyces orientalis* CBS 10300; Lipstark1_1, *Lipomyces starkeyi* Phaff 55–103; Lipsta1_1, *Lipomyces starkeyi* Phaff 78–25; Lipstar1_1, *Lipomyces starkeyi* Phaff 78–24; Lst7851_1, *Lipomyces starkeyi* CBS 7851; Lipdoo1, *Lipomyces doorenjongii* Phaff 78–26; Lipodoor1, *Lipomyces doorenjongii* NRRL Y-27504; Lst8064_1, *Lipomyces starkeyi* CBS 8064; Lipkon1, *Lipomyces kononenkoae* NRRL Y-11553; Yarlip1, *Yarrowia lipolytica* FKP355; Lipmes1, *Lipomyces mesembrius* CBS 7600; Lipchi1, *Lipomyces chichibuensis* CBS 12929; Babin1, *Babjeviella inositovora* NRRL Y-12698; Myxme1, *Myxozyma melibiosi* Phaff 52–87; Lipsuo1, *Kockiozyma suomiensis* NRRL Y-17356; Lipsmi1, *Limtongia smithiae* NRRL Y-17922; Dipuni1, *Dipodascopsis uninucleata* Phaff 50–6; Lipjap1, *Lipomyces japonicus* CBS 7319; Lipoli1, *Lipomyces oligophaga* CBS 7107; Liparx1, *Lipomyces arxii* Phaff 12–163; Diptot1, *Dipodascopsis tothii* CBS 759.85.
