# Supplementary material for: Genome-scale model development and genomic sequencing of the oleaginous clade Lipomyces
Source: Front Bioeng Biotechnol. 2024 Apr 4;12:1356551. doi: 10.3389/fbioe.2024.1356551 (PMC11024372; doi:10.3389/fbioe.2024.1356551)
Supplement: Supplementary file 5 [file DataSheet1.docx]

**Supplemental Figures and Tables.**

*
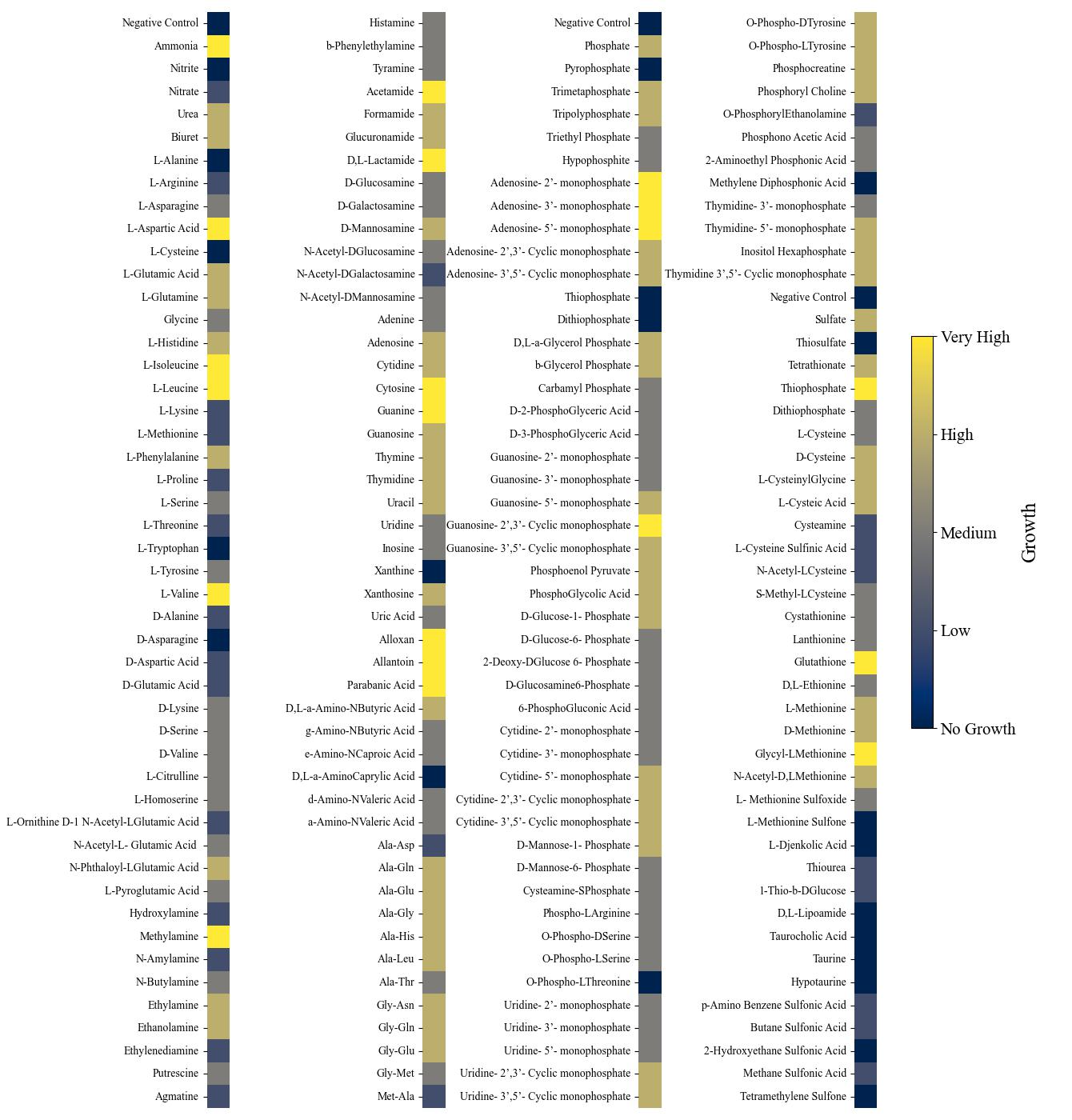
*

**Figure S1.** **Biolog Results from Nitrogen (left two columns), Phosphorus (third column) and Sulfur (last column) sources.** Growth was classified into categories based on the maximum OD_750_ value achieved on each substrate as compared to the negative control for each nutrient source and plate.

**
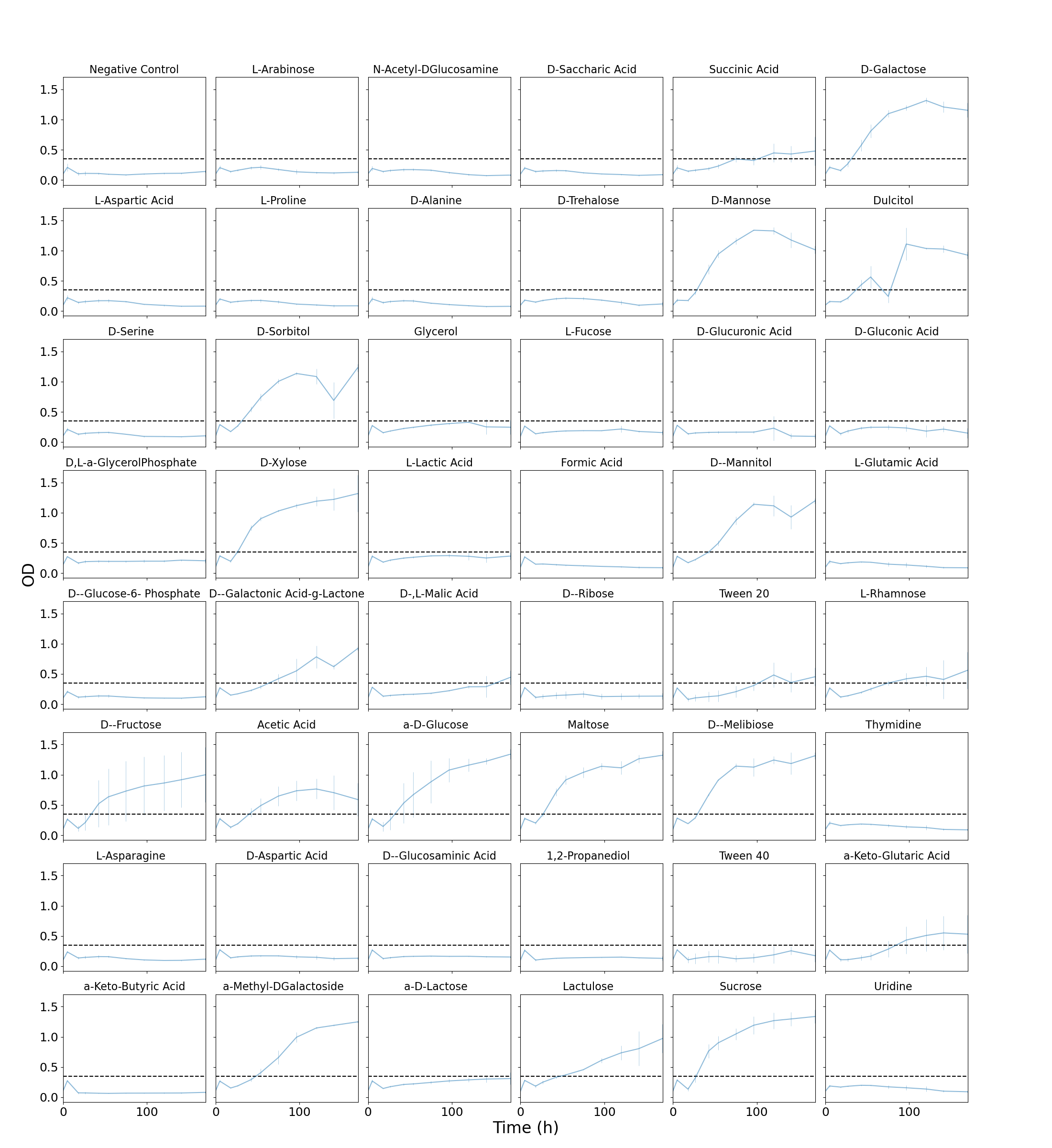
**

**Figure S2. First set of the Biolog Growth Data for carbon sources as measured at 750 nm.** Black dashed line represents the threshold for growth/no growth set based on the negative control value.

**
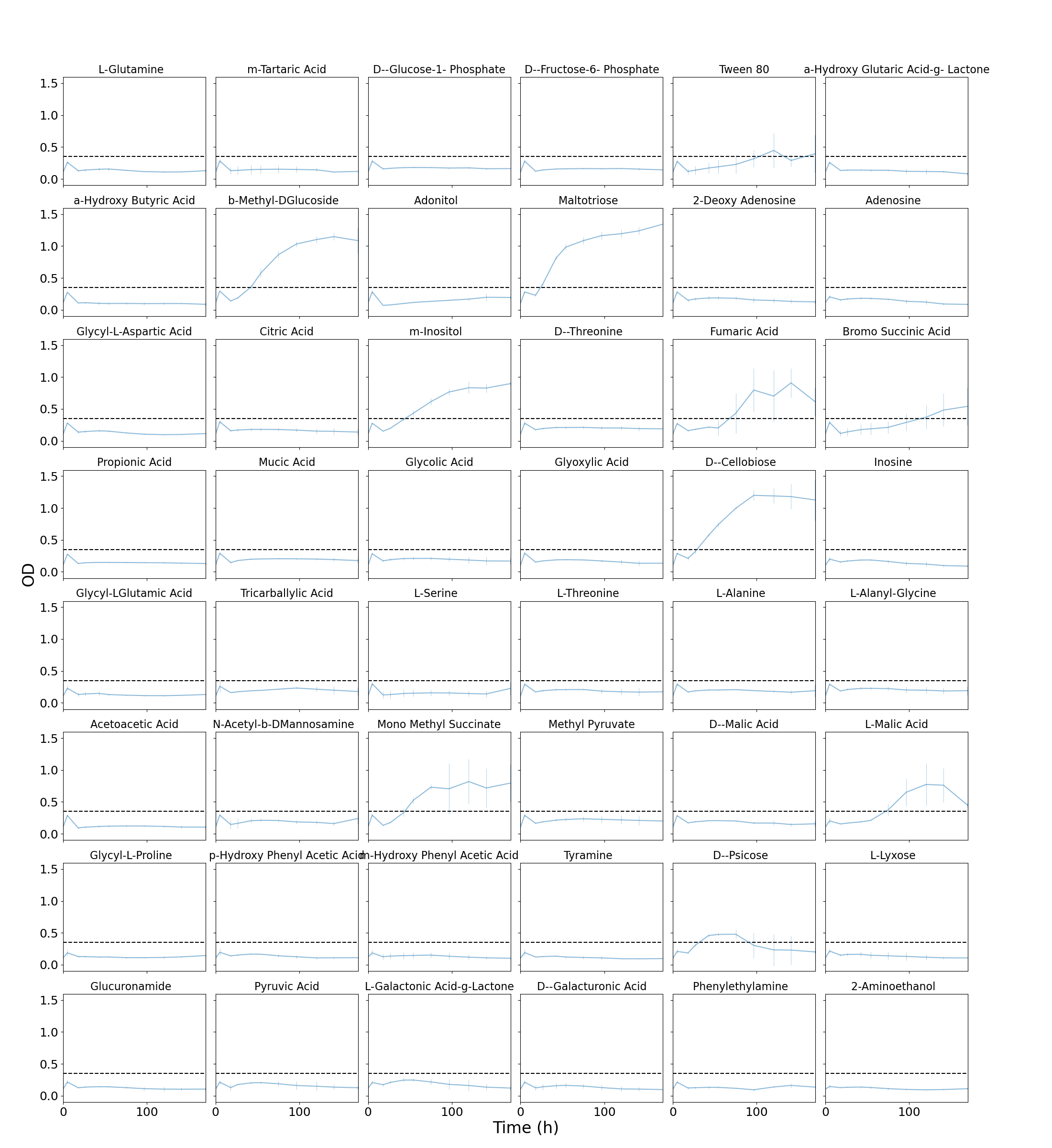
**

**Figure S3. Second set of the Biolog Growth Data for carbon sources as measured at 750 nm.** Blue lines are OD measured at 750 nm. Black dashed line represents the threshold for growth/no growth set based on the negative control value (as displayed in Figure S1).


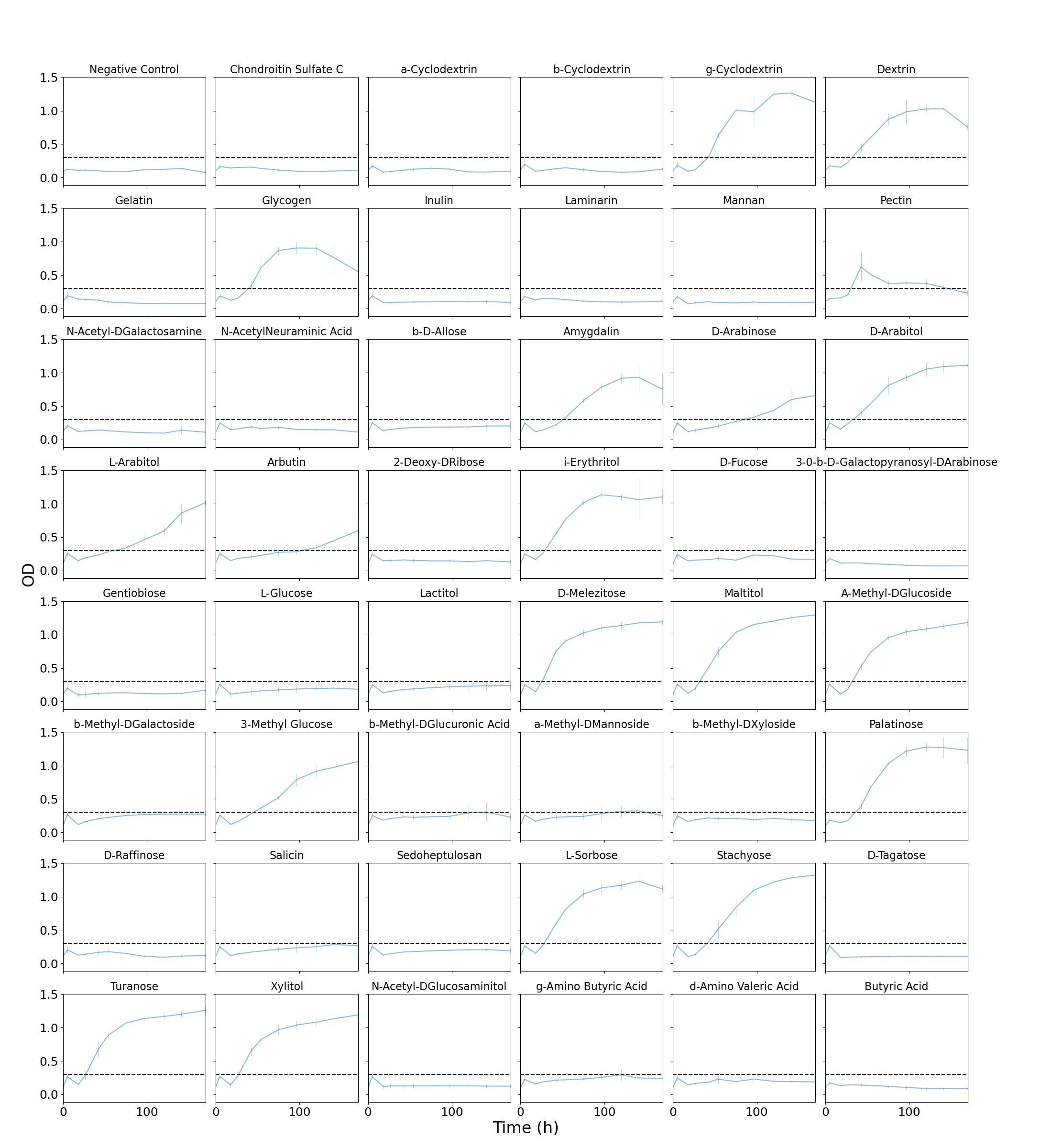


**Figure S4. Third set of the Biolog Growth Data for carbon sources as measured at 750 nm.** Blue lines are OD measured at 750 nm. Black dashed line represents the threshold for growth/no growth set based on the negative control value.

**
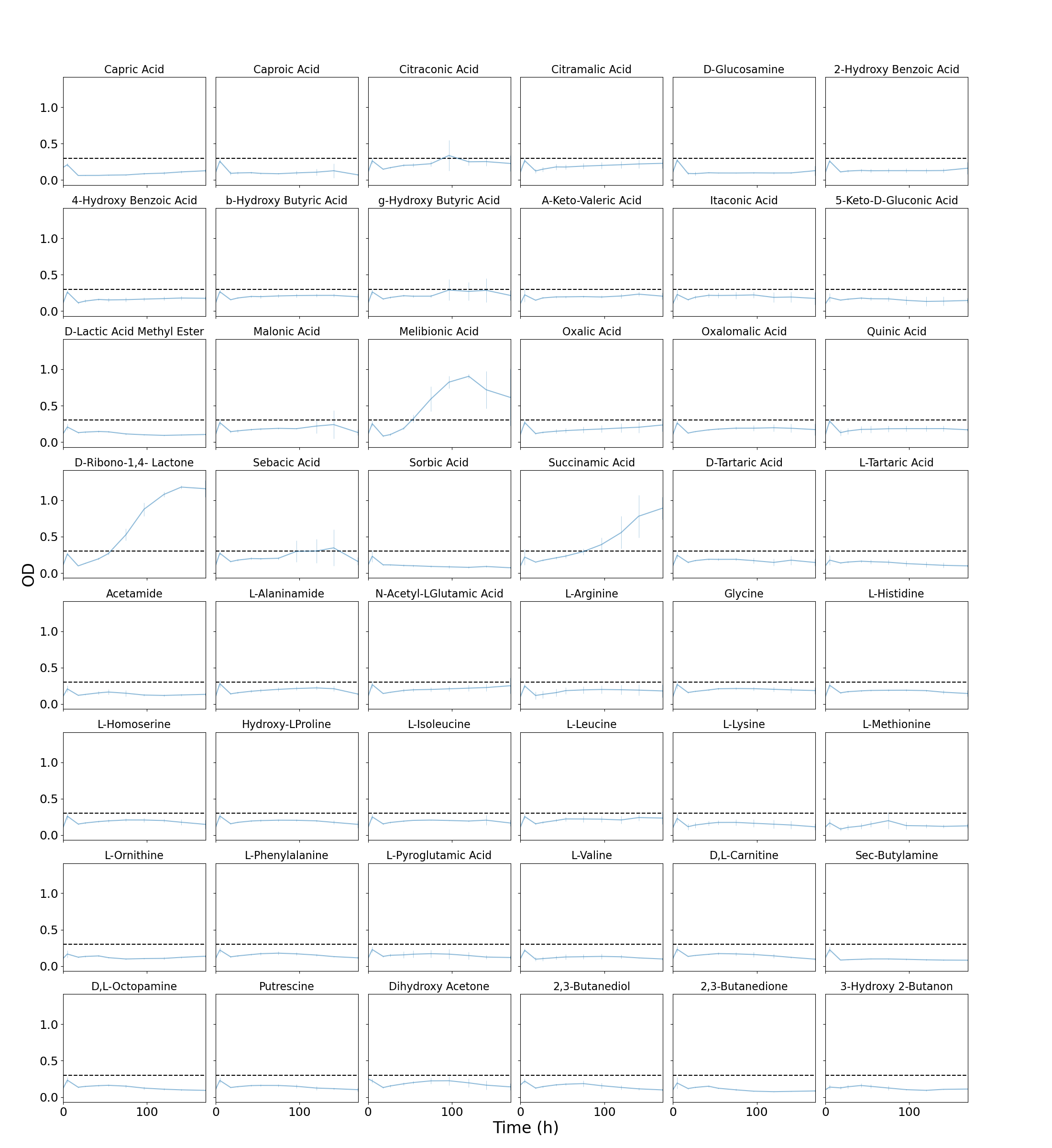
**

**Figure S5. Fourth set of the Biolog Growth Data for carbon sources as measured at 750 nm.** Blue lines are OD measured at 750 nm. Black dashed line represents the threshold for growth/no growth set based on the negative control value (as displayed in Figure S3).

**
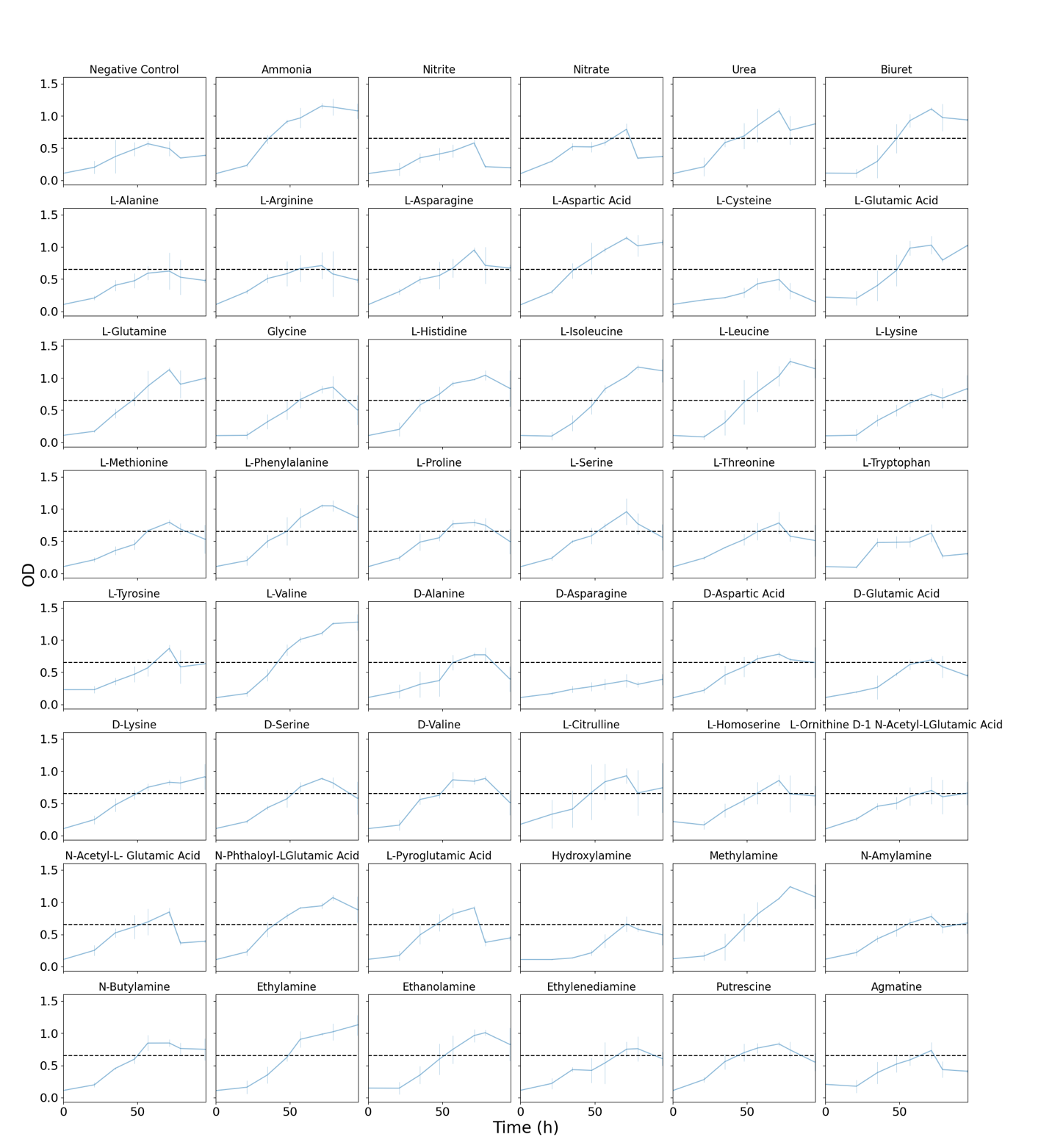
**

**Figure S6. First half of the Biolog Growth Data for nitrogen sources as measured at 750 nm.** Blue lines are OD measured at 750 nm. Black dashed line represents the threshold for growth/no growth set based on the negative control value.


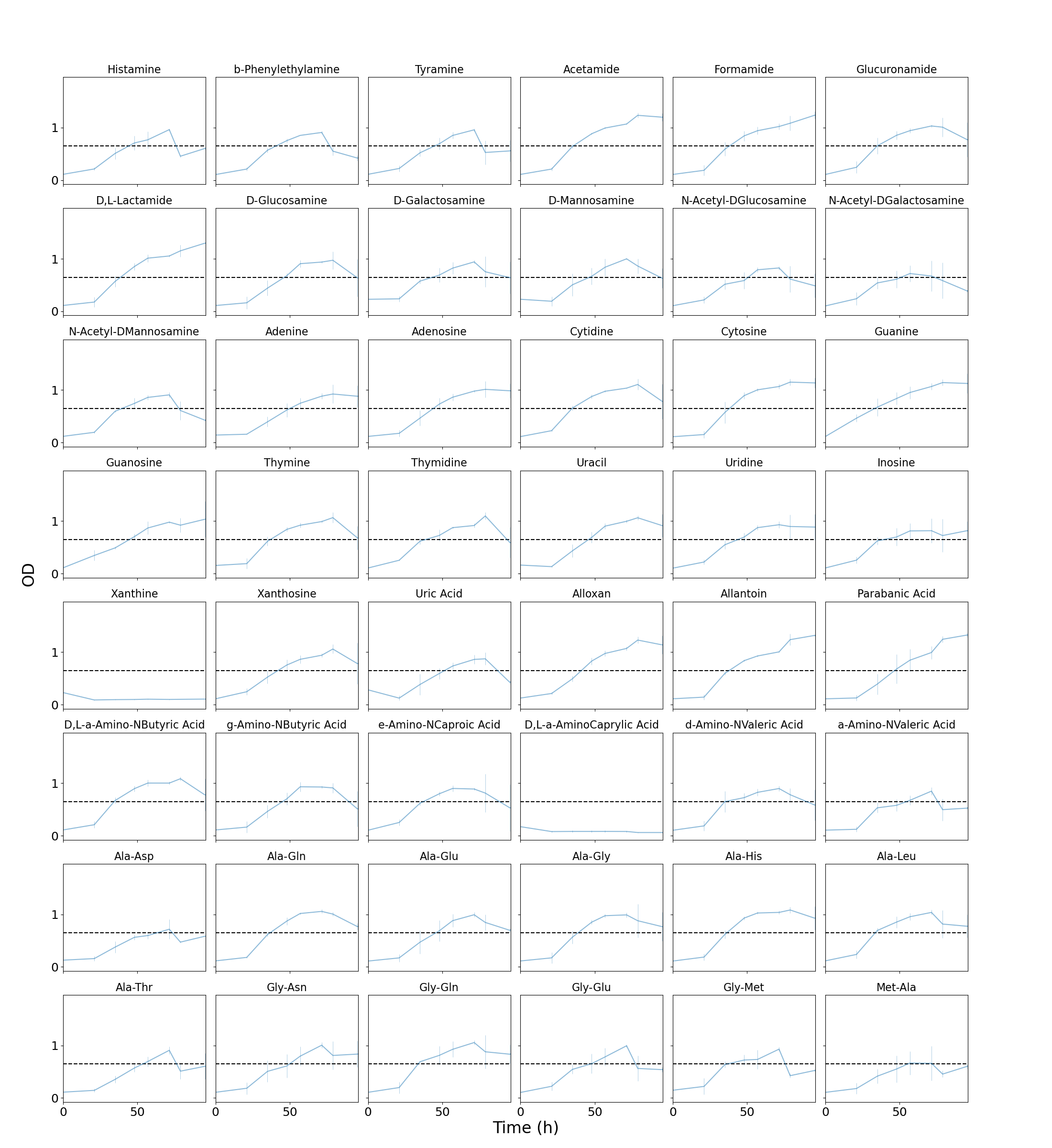


**Figure S7. Second half of the Biolog Growth Data for nitrogen sources as measured at 750 nm.** Blue lines are OD measured at 750 nm. Black dashed line represents the threshold for growth/no growth set based on the negative control value (as displayed in Figure S5).

**
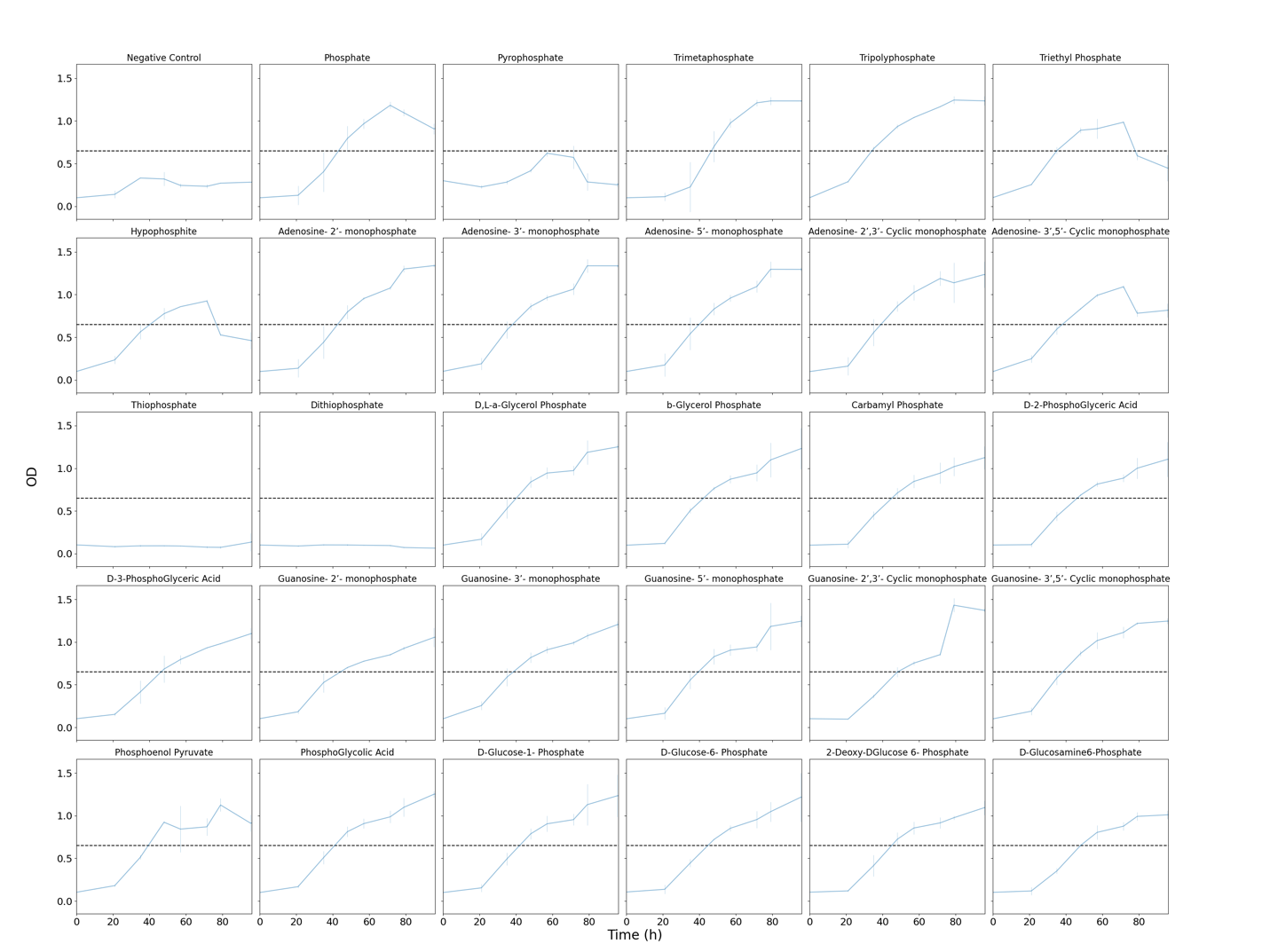
**

**Figure S8. First set of Biolog growth data for phosphorus sources as measured at 750 nm.** Blue lines are OD measured at 750 nm. Black dashed line represents the threshold for growth/no growth set based on the negative control value.


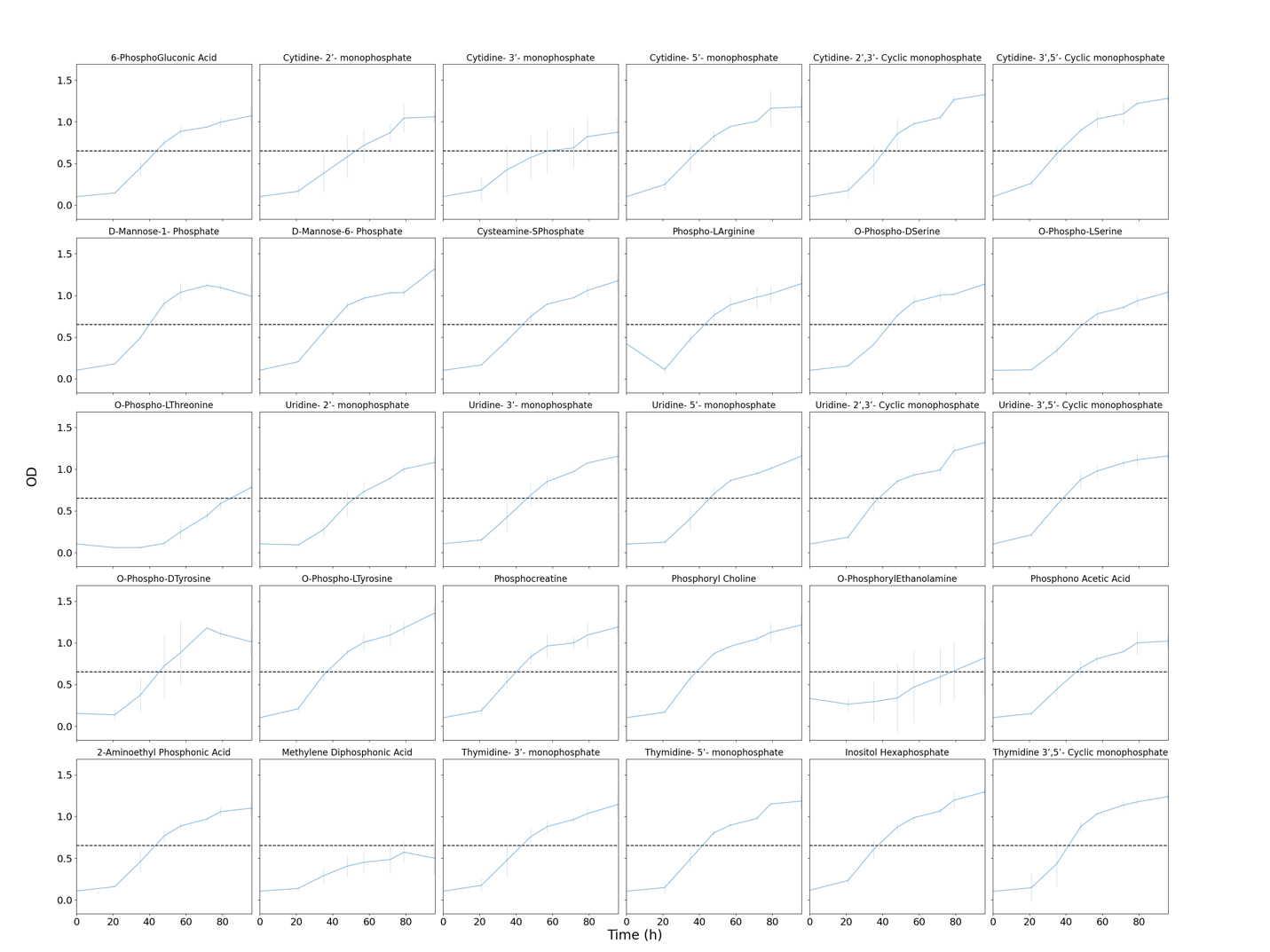


**Figure S9. Second set of Biolog growth data for phosphorus sources as measured at 750 nm.** Blue lines are OD measured at 750 nm. Black dashed line represents the threshold for growth/no growth set based on the negative control value.


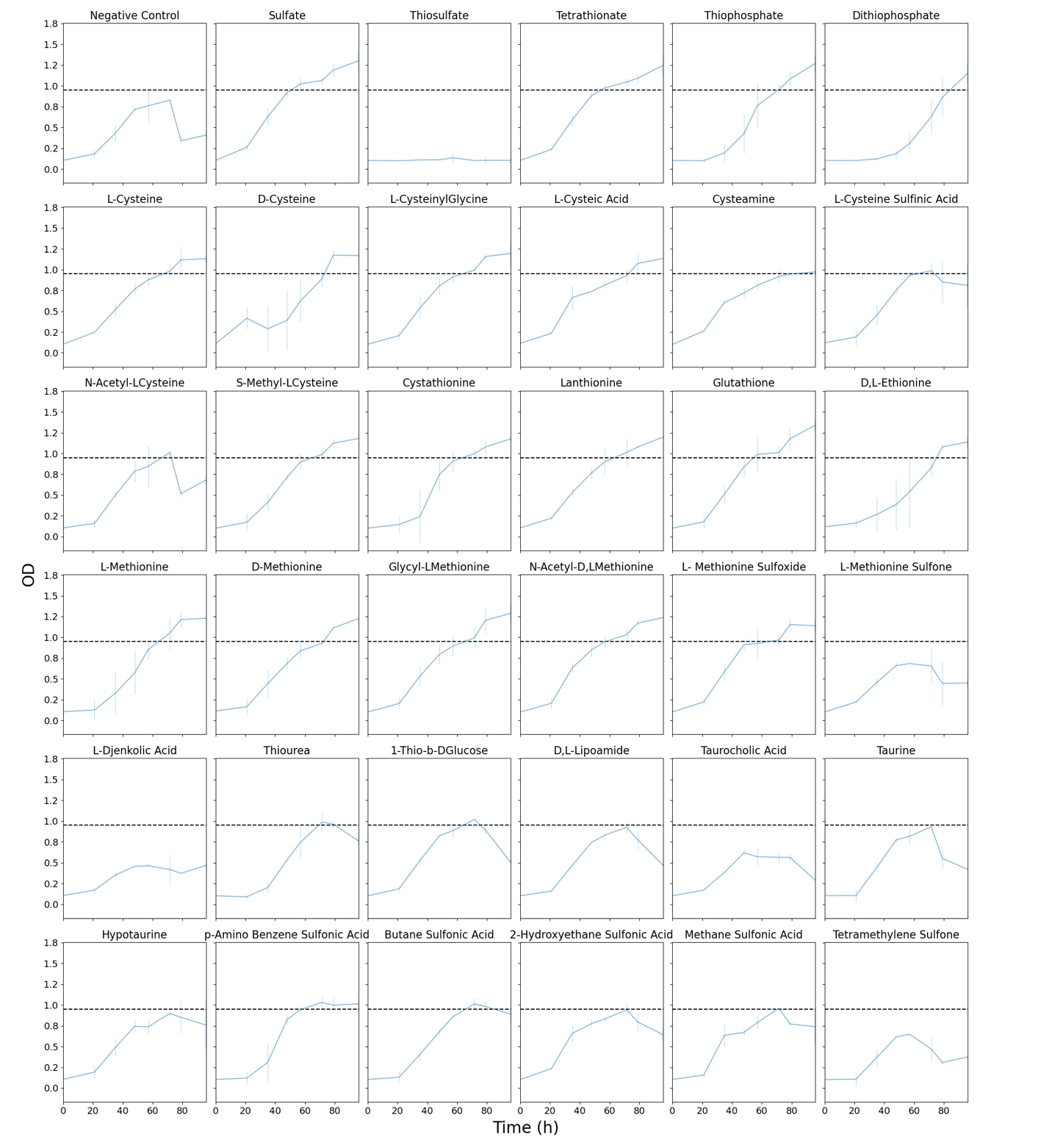


**Figure S10. Biolog Growth Data for sulfur sources as measured at 750 nm.** Blue lines are OD measured at 750 nm. Black dashed line represents the threshold for growth/no growth set based on the negative control value (as displayed in Figure S1).


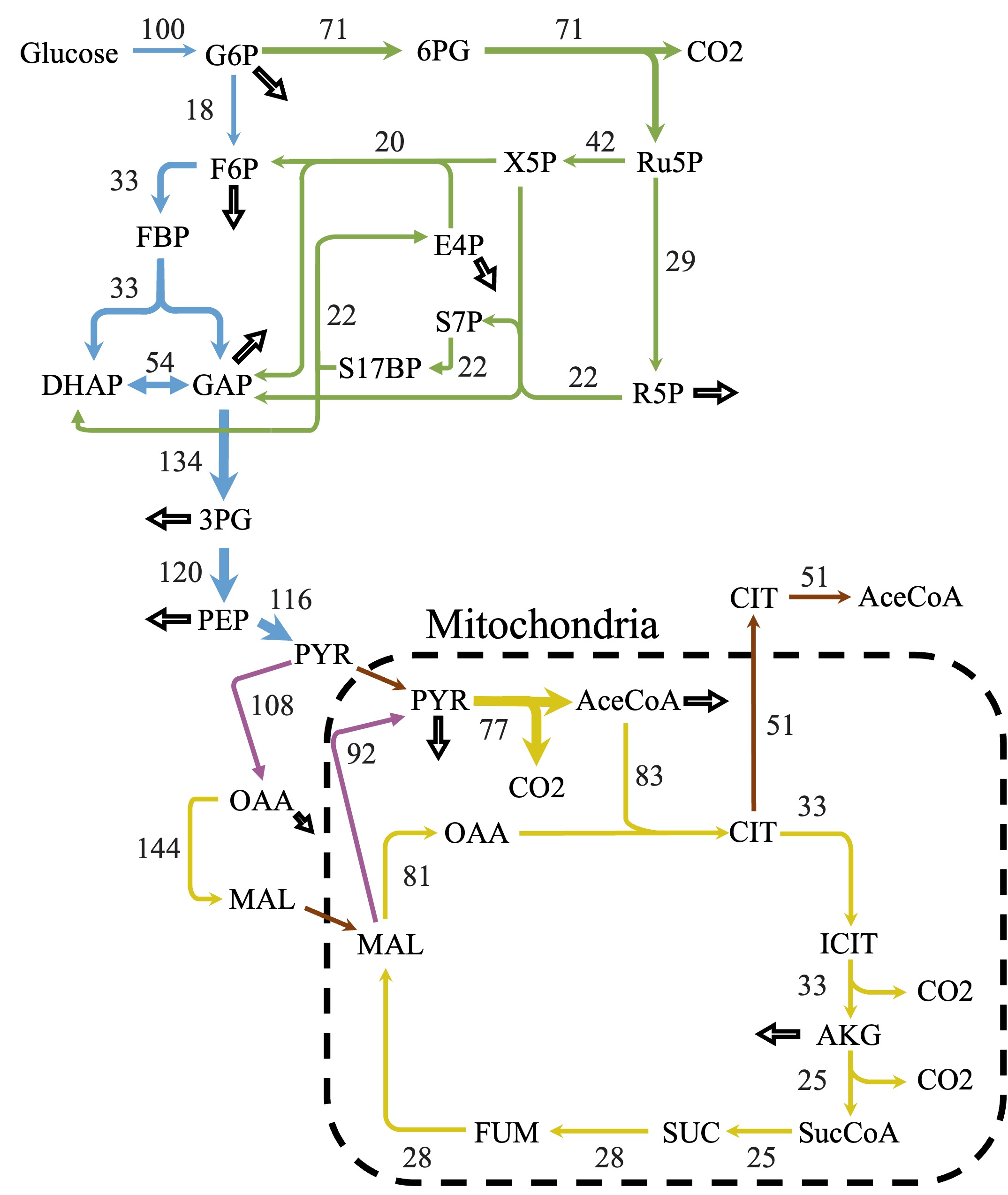


**Figure S11. Predicted metabolic flux map predicted from model iLst996 when redox cycles are blocked.** The central carbon predicted flux values using parsimonious flux balance analysis and with an uptake of 1 mmol/gDCW/h of glucose. Flux values were normalized to a percentage of carbon source uptake. Colors correspond to different metabolic pathways. Blue – glycolysis, Green – pentose phosphate pathway, yellow – citric acid cycle, purple – anaplerotic reactions, brown – transport reactions. Hollow arrows represent biomass drainage reactions.


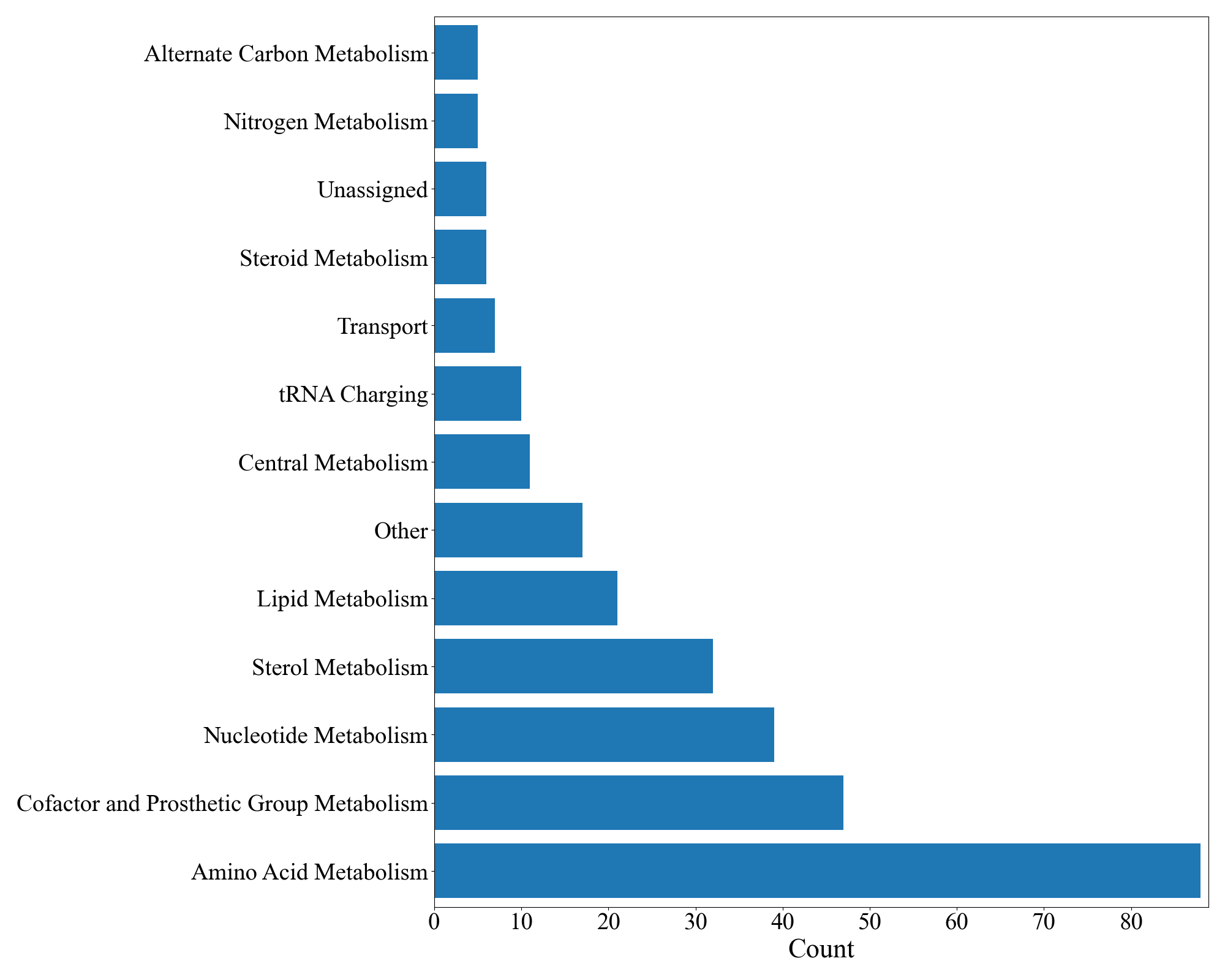


**Figure S12. Pathways of Essential Genes.**


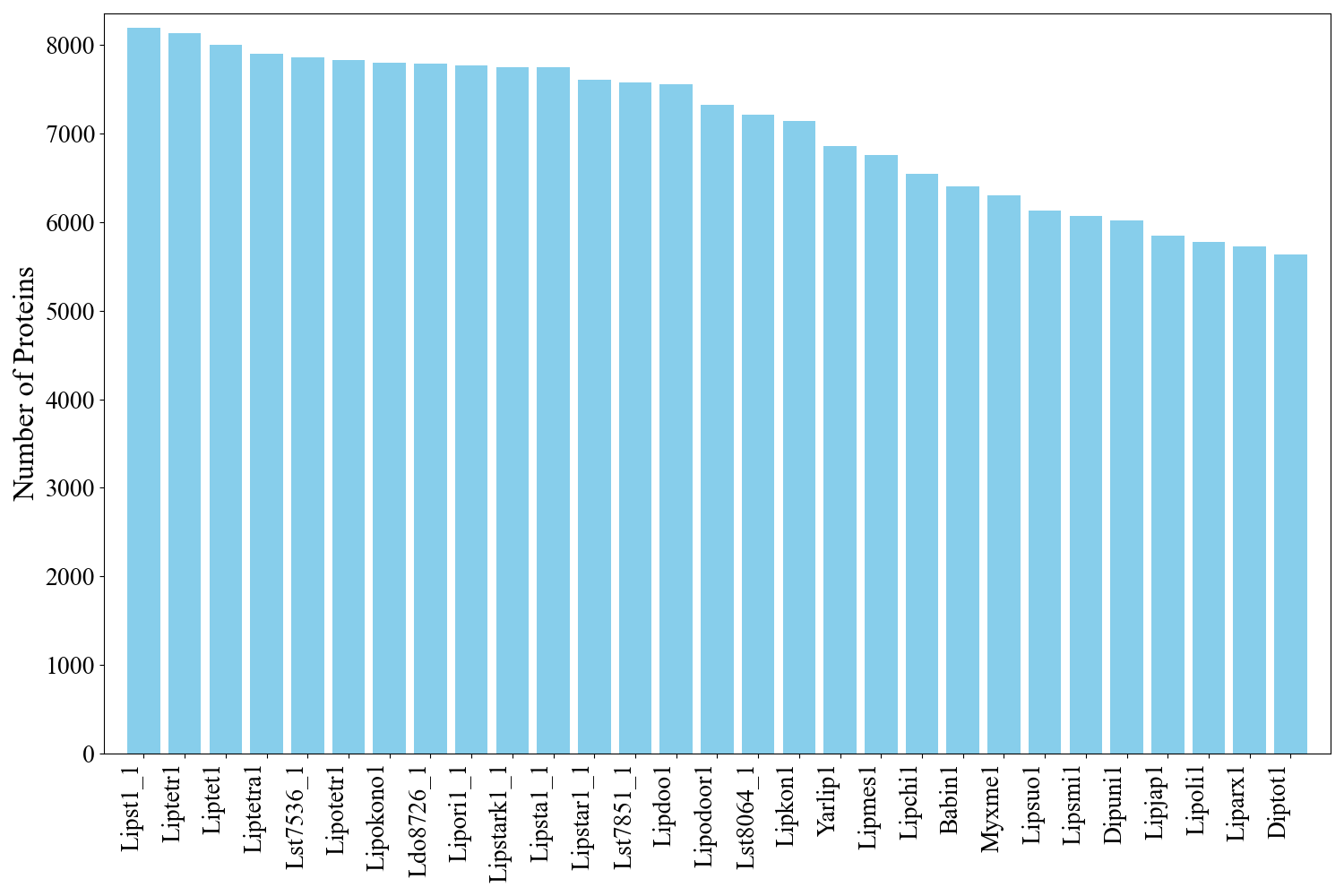


**Figure S13. Protein counts of the orthoMCL analyzed species.**

**
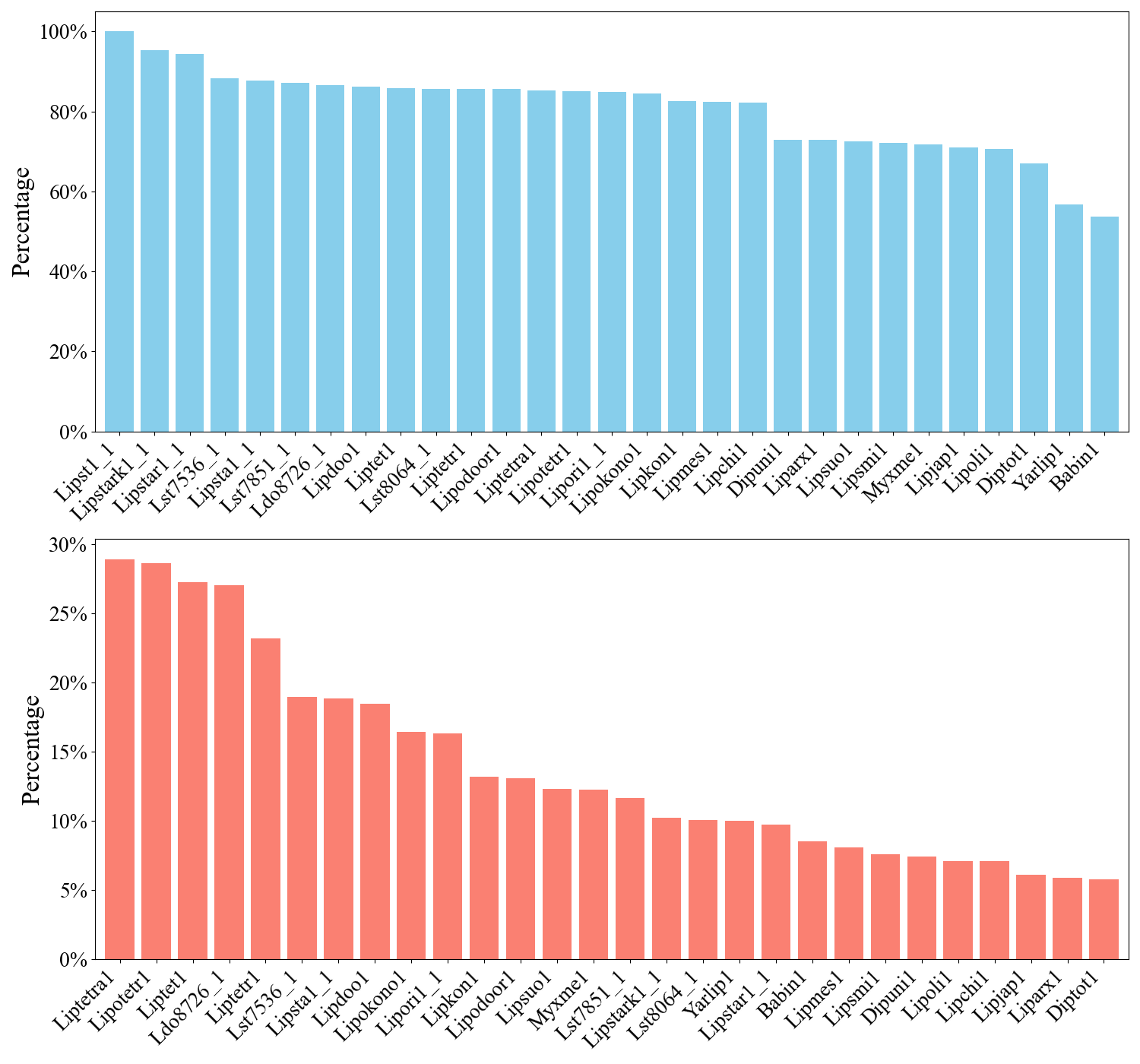
**

**Figure S14. Percent presence of species’ genes in groups with a *L. starekeyi* NRRL Y-11557 gene (top) and those without (bottom).**

**
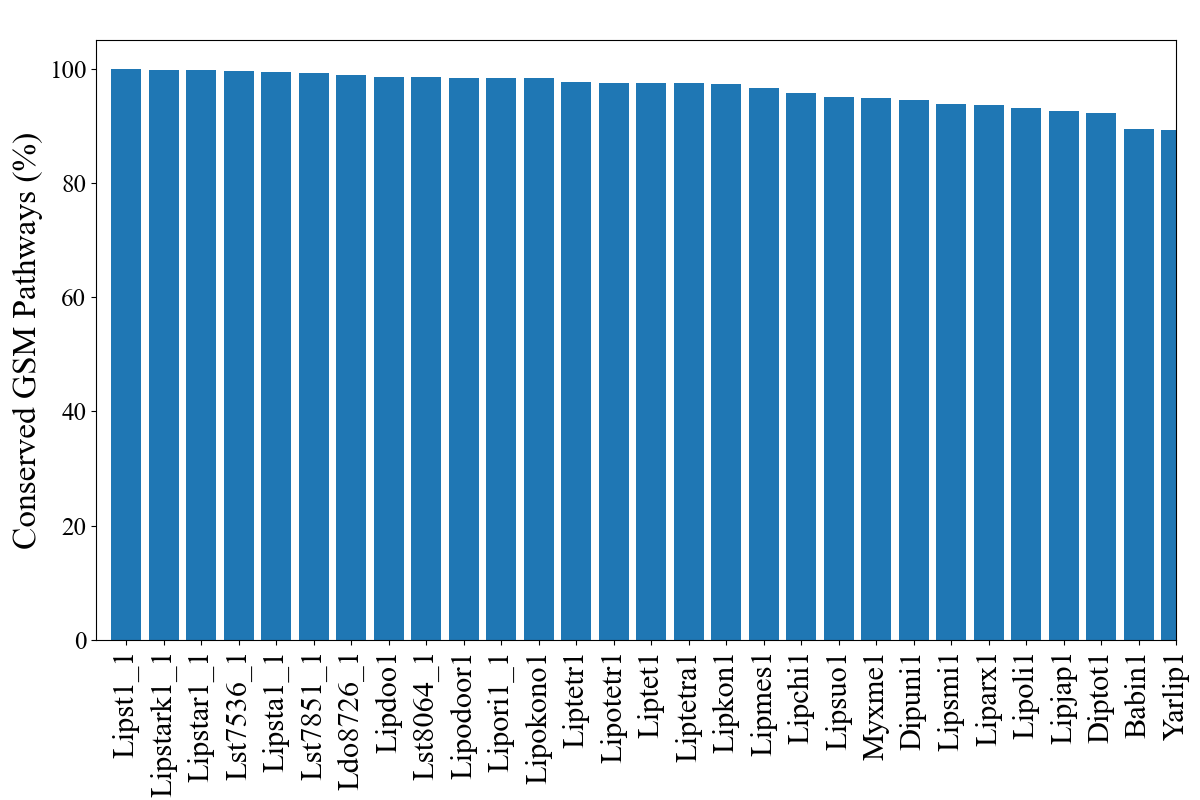
**

**Figure 15. Precent of conserved GSM reactions in each organism.** The percentage represents the number of reactions in the GSM for which there is an ortholog gene present in the specified organism.

**Tables.**

**Table S1. Updated *L. starkeyi* Biomass Equation**

| Reaction ID |  |
| --- | --- |
| BIOMASS_Ls | '0.6542899047921068 13BDglcn_c + 0.12116467064111869 16BDglcn_c + 0.0008767124745935498 5mthf_c + 0.45552 alatrna_c + 0.141799 argtrna_c + 0.132431 asntrna_c + 0.204341 asptrna_c + 96.233892 atp_c + 0.0016522920994288411 btn_m + 0.0005685306148572357 ca2_c + 0.001224527478154046 camp_c + 0.021186648694289174 chitin_c + 1.1e-05 clpn_LS_m + 0.0005261641507693166 coa_c + 0.045479 ctp_c + 0.0003587482846154432 cu2_c + 0.040268 cystrna_c + 0.003483 datp_c + 0.003166 dctp_c + 0.00278 dgtp_c + 0.003587 dttp_c + 0.005033 ergst_r + 1e-05 ergstest_LS_r + 0.0005131808795165673 fad_c + 0.00040794804936270397 fe2_c + 0.00040794804936270397 fe3_c + 0.115471 glntrna_c + 0.199788 glutrna_c + 0.3540121239284534 glycogen_c + 0.442117 glytrna_c + 0.0013119937265936208 gthrd_c + 0.050529 gtp_c + 92.341901 h2o_c + 0.0004721810755605166 hemeA_m + 0.060743 histrna_c + 0.201923 iletrna_c + 0.4002284696078455 k_c + 0.293113 leutrna_c + 0.0021231065148574896 lipopb_m + 0.16701 lystrna_c + 0.5520903768009253 mannan_r + 0.065101 mettrna_c + 0.0421723983491937 mg2_c + 0.0008828624451869573 mlthf_c + 0.00041478135002204577 mn2_c + 0.017834231390816097 na1_c + 0.0006067970985495497 nad_c + 0.0005432474024176711 nadp_c + 6e-06 pa_LS_r + 0.000172 pc_LS_r + 0.000107 pe_LS_r + 0.108322 phetrna_c + 0.20874 protrna_c + 4.3e-05 ps_LS_r + 0.010348 psphings_r + 4.2e-05 ptd1ino_LS_r + 0.0044580453501545745 ptrc_c + 0.0016399921582420259 pydx5p_c + 0.0005056642487912913 q9_m + 0.0006764967652748358 ribflv_c + 0.40151 sertrna_c + 0.0027107703715608823 spmd_c + 0.0009067789974946536 thf_c + 0.0009518787818463092 thmpp_c + 0.226918 thrtrna_c + 0.015970790301013598 tre_c + 0.00182 triglyc_LS_r + 0.025613 trptrna_c + 0.077745 tyrtrna_c + 0.050281 utp_c + 0.273459 valtrna_c + 0.00034849833362643056 zn2_c + 0.005191 zymst_r + 1e-05 zymstest_LS_d --> 96.183832 adp_c + 96.183832 h_c + 96.183832 pi_c + 2.000000000002e-06 ppi_c' |

**Table S2. Lipid yields on different carbon sources (uptake set at 1 mmol/gDCW/h), at 10% biomass yields.**

|  | Maximum predicted growth rate (h^-1^) | Maximum theoretical lipid yield (g TAG/g substrate) | Maximum theoretical lipid yield (Cmol TAG/ Cmol substrate) |
| --- | --- | --- | --- |
| Glucose | 0.0889 | 0.30 | 0.57 |
| Xylose | 0.0731 | 0.29 | 0.55 |
| Sucrose | 0.1786 | 0.31 | 0.57 |
| Gluconate | 0.0814 | 0.25 | 0.52 |
| Maltose | 0.1786 | 0.31 | 0.57 |
| Glycerol | 0.0511 | 0.33 | 0.65 |
| Acetate | 0.0227 | 0.25 | 0.47 |
| Palmitic acid | 0.2691 | 0.83 | 0.84 |
